# Modeling the global effect of the basic-leucine zipper transcription factor 1 (bZIP1) on nitrogen and light regulation in Arabidopsis

**DOI:** 10.1186/1752-0509-4-111

**Published:** 2010-08-12

**Authors:** Mariana Obertello, Gabriel Krouk, Manpreet S Katari, Suzan J Runko, Gloria M Coruzzi

**Affiliations:** 1 Center for Genomics and Systems Biology, Department of Biology, New York University. 100 Washington Square East, 1009 Main Building, New York, NY 10003, USA; 2 Institut de Biologie Intégrative des Plantes, UMR 5004, Biochimie et Physiologie Moléculaire des Plantes, Agro-M/CNRS/INRA/SupAgro/UM2, Montpellier, France; 3 Instituto de Ingeniería Genética y Biología Molecular (INGEBI-CONICET), Vuelta de Obligado 2490 piso 2, C1428ADN Buenos Aires, Argentina

## Abstract

**Background:**

Nitrogen and light are two major regulators of plant metabolism and development. While genes involved in the control of each of these signals have begun to be identified, regulators that integrate gene responses to nitrogen and light signals have yet to be determined. Here, we evaluate the role of bZIP1, a transcription factor involved in light and nitrogen sensing, by exposing wild-type (WT) and bZIP1 T-DNA null mutant plants to a combinatorial space of nitrogen (N) and light (L) treatment conditions and performing transcriptome analysis. We use ANOVA analysis combined with clustering and Boolean modeling, to evaluate the role of bZIP1 in mediating L and N signaling genome-wide.

**Results:**

This transcriptome analysis demonstrates that a mutation in the bZIP1 gene can alter the L and/or N-regulation of several gene clusters. More surprisingly, the bZIP1 mutation can also trigger N and/or L regulation of genes that are not normally controlled by these signals in WT plants. This analysis also reveals that bZIP1 can, to a large extent, invert gene regulation (e.g., several genes induced by N in WT plants are repressed by N in the bZIP1 mutant).

**Conclusion:**

These findings demonstrate that the bZIP1 mutation triggers a genome-wide de-regulation in response to L and/or N signals that range from i) a reduction of the L signal effect, to ii) unlocking gene regulation in response to L and N combinations. This systems biology approach demonstrates that bZIP1 tunes L and N signaling relationships genome-wide, and can suppress regulatory mechanisms hypothesized to be needed at different developmental stages and/or environmental conditions.

## Background

Nitrogen (N) and light (L) are two important signals that regulate plant growth and development. Microarray studies have been used to investigate the genome-wide effects of regulatory interactions of signals like N, carbon (C) and L, as follows: C and N signaling [1-3], C and L signaling [4,5], C and circadian rhythm [6,7]. More recently, a study explored genome-wide effects of all combinations of C, L, N interactions in two different organs [8]. However, very little is known of the regulatory networks involved in the perception and transduction of N and L signals and their cross-talk [9]. A mechanism by which N and L signals exert their effects on plants is through their ability to affect the expression of a large number of genes. In fact, a number of transcription factors associated with changes in gene expression by N and L have been identified; interestingly, AtbZIP1 has been hypothesized to be one of them [10-13].

The basic-region leucine zipper (bZIP) transcription factor family is represented by multiple genes, encoding proteins that contain a basic region involved in DNA binding and nuclear import, and a leucine zipper dimerization domain [14,15]. It is becoming clear that plant bZIP factors are regulated by post-translational mechanisms affecting their DNA binding and transcriptional properties, their stability, and their capacity to form homodimers and heterodimers, and to interact with non-bZIP proteins [14]. These mechanisms enable a rapid and often reversible adaptation of bZIP activities in response to endogenous and environmental cues [14]. The *Arabidopsis thaliana *genome encodes approximately 75 predicted bZIP factors [16,17]. Like other transcription factor genes in plants, members of the bZIP transcription factor family are expressed in an organ-specific manner [18], and have been shown to regulate diverse biological processes such as stimulus-response [19,20], cell cycle specificity [21], control seed storage and maturation [22], pathogen defense and flower development [16]. It has also been recently shown that the activity of several bZIP transcription factors (including bZIP1) is partially mediated by KIN10, a kinase that is a central integrator of a transcription network involved in plant stress response and energy signaling [12]. Most recently, Kang et al. [23] studied the role of AtbZIP1 in sugar-mediated gene expression using a reverse genetic analyses. Their results indicate that AtbZIP1 acts as a negative regulator of early seedling growth in the absence of exogenous sugars in the culture medium, implicating a role of AtbZIP1 in sugar-mediated gene expression.

To identify the potential role of bZIP1 as an integrator of N and L signaling, this study identifies and characterizes the molecular defects of a bZIP1 T-DNA mutant on a genome-wide scale in the context of combinatorial treatments of N and L. Several models describing the role of bZIP1 in the genome-wide integration of these signals are derived from this analysis.

## Results

**Figure 1:**
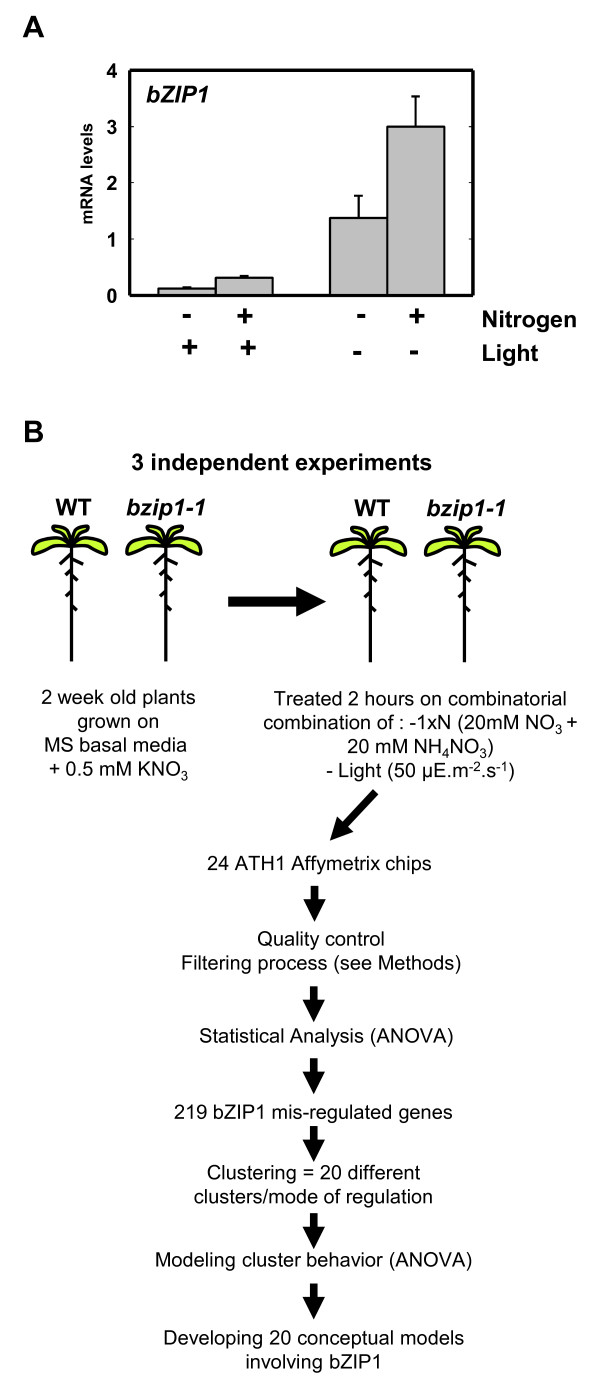
**Experimental design for systematic analysis of N and/or L-regulation via bZIP1**. (A) Quantification of *bZIP1 *mRNA levels in Col-0 plants. Transcript levels are determined by real-time PCR and are shown as relative to expression of Clathrin gene. Values are the mean ± SE from three biological replicates. (B) Schematic diagram of the data mining approach used in this study. Briefly, WT siblings and *bzip1-1 *T-DNA mutant plants are treated with combinatorial conditions varying light and nitrogen. Genome-wide analysis using ATH1 Affymetrix chips has been used in order to quantify mRNA levels. Modeling of microarray data, using ANOVA/clustering procedure (detailed in Methods), enables the identification of genes for which bZIP1 is involved in mediating the effects of N and/or L signal integration.

Several previous studies, including one from our laboratory, indicate that the *bZIP1 *transcription factor is regulated in response to nitrogen (N) and light (L) treatments [10-12]. To further study the role of bZIP1 in mediating the integration of N and L signaling, we studied the effect of a bZIP1 mutation in contrasted nutritional conditions of N and L combinatorial treatments. To interpret the results, we developed an analysis involving reverse genetics and statistical modeling of transcriptome data generated from different treatments that systematically vary nitrogen and light as input signals (Figure 1), as described below.

### Characterization of *bZIP1 *regulation and isolation of T-DNA knock-out mutants

We tested conditions of pre-treatment and treatment that optimized *bZIP1 *regulation in response to nitrogen and light treatments. Following this protocol, 14-day-old Arabidopsis seedlings grown in low N conditions (0.5 mM KNO_3_), were treated with nitrogen concentrations found in standard MS media (40 mM NO_3 _and 20 mM NH_4_, referred herein as 1xN) for 2 hrs in either light- or dark-adapted growth conditions (see Methods). The 1xN treatment consists of the same N source found in standard MS salts, which is the established standard amount of N for plant growth [24], and was successfully used before to identified inorganic and organic N responses in Arabidopsis [10]. Thus, plants were subject to four different nitrogen and/or light treatments (-N-L (as control); +N-L; -N+L; +N+L). Quantification of *bZIP1 *mRNA in wild-type Columbia ecotype (Col-0) plants, confirmed that *bZIP1 *mRNA is induced by N-treatment and repressed by L-treatment as described below (Figure 1A). In wild-type Col-0 plants, N-treatment led to an equal induction of *bZIP1 *mRNA in either absence or presence of light: +N-L/-N-L = 2.17 fold; +N+L/-N+L = 2.60 fold (2-way ANOVA nitrogen effect *p *< 0.05). Similarly, L-treatment led to an equal repression of *bZIP1 *in either the absence or presence of N; -N-L/-N+L = 11.65 fold; +N-L/+N+L = 9.74 fold (2-way ANOVA light effect *p *< 0.0001). This result demonstrates that *bZIP1 *mRNA levels are controlled by N or L signaling, but that these signals do not interact in the control of *bZIP1 *mRNA accumulation (2-way-ANOVA interaction factor between N and L is non-significant). In other words, N and L act independently to control *bZIP1 *mRNA accumulation.

**Figure 2:**
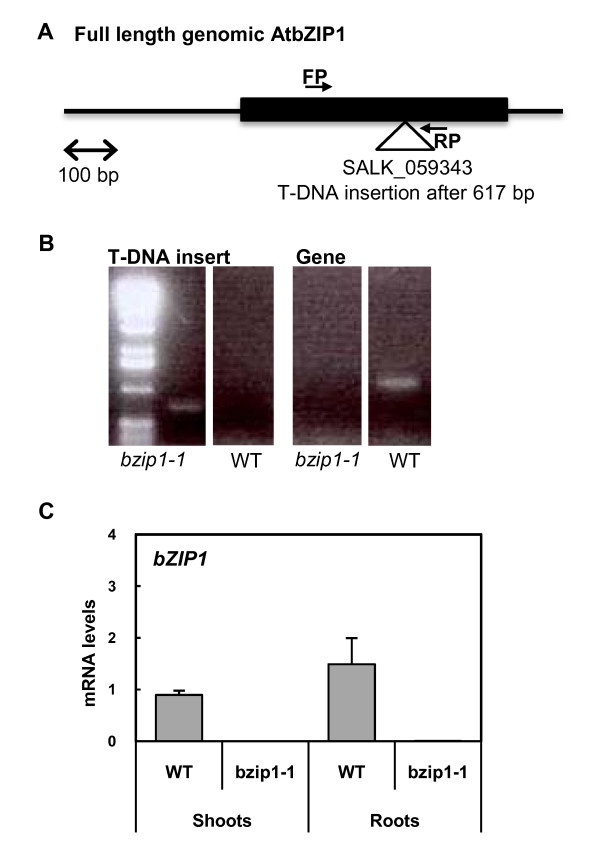
**Molecular characterization of *bzip1-1***. (A) Relative position of T-DNA insertion in bZIP1 gene and primers used for the real-time PCR (FP: forward primer; RP: reverse primer). (B) Comparative PCR of the flanking region T-DNA insertion site. (C) Expression of *bZIP1 *mRNAs in WT siblings and *bzip1-1 *plants. Transcript levels are determined by real-time PCR and are shown as relative to expression of Clathrin gene. Values are the mean ± SE from three biological replicates.

In order to investigate the role of bZIP1 in the control of gene expression in response to N and L treatments, we obtained a T-DNA mutant knock-out Arabidopsis line (*bzip1*-*1*) (see Methods), which contains a T-DNA insertion in its single exon (Figure 2A). We characterized bZIP1 mutant lines for homozygous for the T-DNA insert. We also characterized a WT sibling (WT-sib) derived from a backcross of a heterozygous line for the bZIP1 T-DNA mutation, as identified by PCR analysis on genomic DNA (Figure 2B). Quantification of *bZIP1 *mRNA levels by RT-PCR, revealed that transcripts of *bZIP1 *are undetectable in the homozygous *bzip1*-*1 *T-DNA mutant in either shoots or roots (Figure 2C). To insure that the changes in global gene regulation were due to the deletion in the bZIP1 gene, we compared the expression values of *bZIP1 *mRNA in the *bzip1*-*1 *T-DNA mutant to a WT sibling, as described below.

### Genomic microarray analysis of wild-type and *bzip1-1 *T-DNA mutant seedlings

The *bzip1-1 *T-DNA mutant and WT-sib plants were subjected to the systematic combination of N and L conditions described above (see Figure 1B) in three independent experiments (biological triplicates). RNA obtained from these samples was subjected to transcriptomic analysis using Arabidopsis ATH1 Affymetrix chips (results deposited as GEO accession number GSE21601). Three-way ANOVA (factors being: Light = L, Nitrogen = N, Genotype = G) was used to detect genes for which Genotype factor has an effect on its own, or in combination with either L, N or both (for further details see Methods). This approach detected 4,920 L-regulated genes; 720 N-regulated genes; 114 G-regulated genes; 194 L*N-regulated genes; 56 N*G-regulated genes, 33 L*G-regulated genes; 33 N*L*G-regulated genes (for the complete lists of these groups see Additional file 1). The union of these four gene lists having G (genotype) as an influential factor corresponds to 219 distinct genes (including bZIP1 itself) whose regulation is affected by the *bzip1-1 *mutation (Additional file 2). This analysis shows that a *bZIP1 *mutation affects N and L regulation to the same extent, based on the number of mis-regulated genes (for a gene by gene histogram of regulation please refers to Additional file 3).

### The *bzip1-1 *mutation triggers different modes of mis-regulation

**Figure 3:**
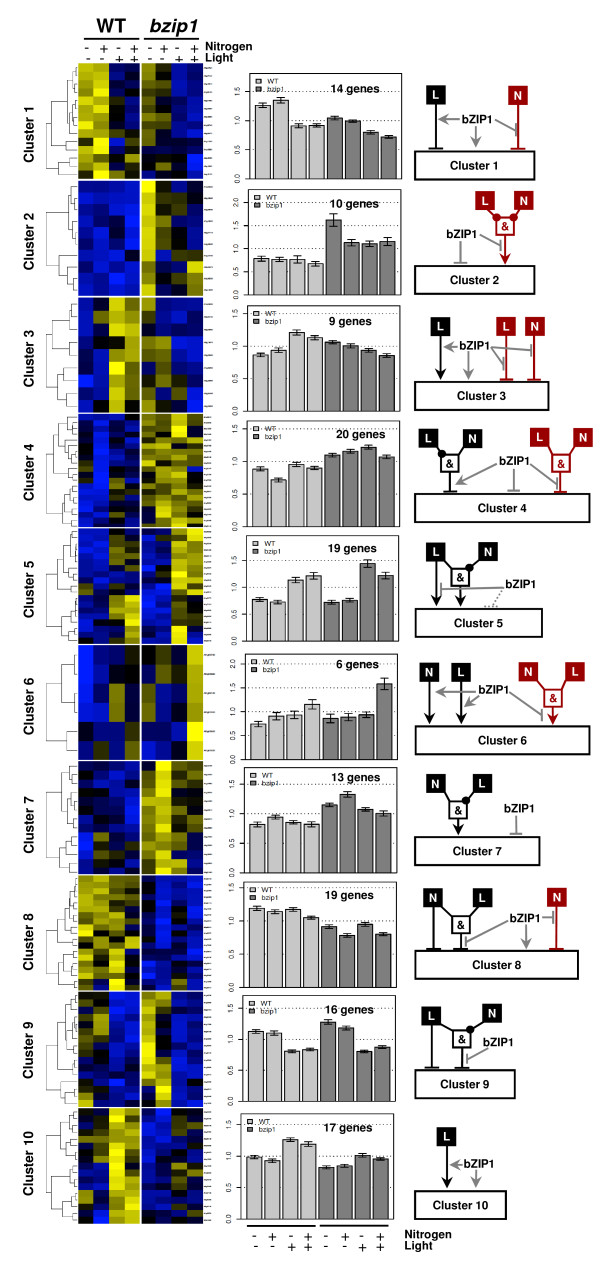
**The *bzip1-1 *mutation triggers different modes of de-regulation in response to N and L treatments (clusters 1 to 10)**. ANOVA identified 219 genes misregulated in *bzip1-1 *compared to a WT sibling that were then used for cluster analysis (see Methods). A heatmap is presented for each cluster, with the regulation of any single gene in it and the histogram is a centroid plot of the whole cluster expression. The whole cluster has been used in order to run a second ANOVA to detect the effect of the signals and the *bzip1-1 *mutation (Additional file 4). The results of the second ANOVA, together with the analysis of the centroid histograms, allowed us to build a conceptual model of the role of the bZIP1 gene for each cluster/mode of de-regulation. As in Boolean logic, a black circle drawn on an edge represents the negation of the signal (for example in cluster 17: "No light and no nitrogen induces cluster expression"). To simplify the interpretation of the figure, when the logic gate uncovered using the *bzip1-1 *mutant plants was different from the one derived for the WT-sib, the Boolean model has been colored in red (to right side of the Boolean conceptual models).

**Figure 4:**
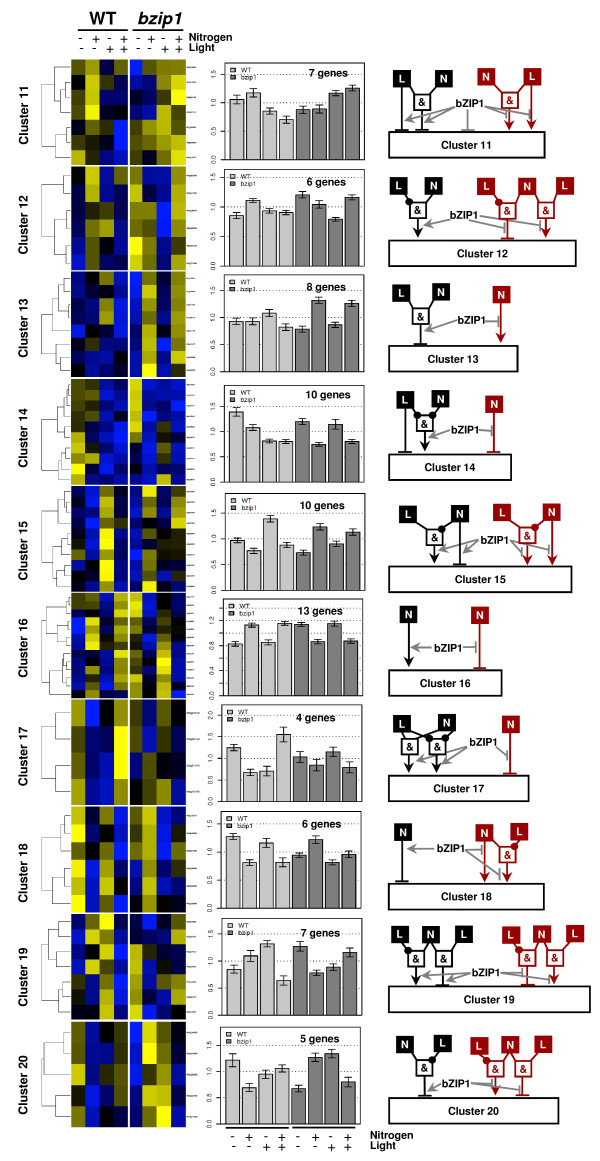
**The *bzip1-1 *mutation triggers different modes of de-regulation in response to N and L treatments (clusters 11 to 20)**. Idem Figure 3

With the aim of gaining further insight into the role of bZIP1 in the regulation of gene expression in response to N *and *L, we clustered the expression of the 219 genes whose response to N and/or L is misregulated in the *bzip1-1 *mutant, compared to its WT sibling (see Methods for details). This analysis generated 20 gene clusters ranging between 4 and 20 genes per cluster (clusters 1 to 10 in Figure 3 and clusters 11 to 20 in Figure 4). The analysis of these gene clusters indicates that the *bzip1-1 *mutation triggers at least four different classes of gene expression deregulation as follows:

In the first class misregulated genes, in four cases (clusters #1, 6, 9, 10) the *bzip1-1 *mutation attenuates signal regulation. All of these clusters have in common an attenuation of L signaling by the bZIP1 mutation. This demonstrates that bZIP1 is directly or indirectly involved in sustaining gene expression modulation by light, as confirmed by previous studies [12].

Unexpected, all clusters in the second class of genes (clusters #3, 11, 15, 16, 18) share the property that the *bzip1-1 *mutation triggers a regulation of gene expression by the N or L signals that is inverse to the WT-sib response. For instance, the 13 regulated genes in cluster #16 are *induced *by N in the WT-sib, while in the *bzip1-1 *mutant, they are *repressed *by N treatment. This observation leads to the tentative hypothesis that bZIP1 is involved in a yet-to-be-defined "switching" system involved in modifying gene response to nutritional cues, perhaps depending on environmental conditions or developmental stages.

This analysis also shows that a third class of genes (clusters #2, 5, 8, 13, 14, 19) encompasses genes for which the *bzip1-1 *mutation triggers a new or hidden regulation. One striking example of this is cluster #2, for which genes are not regulated by N and L in WT-sib. However, when *bZIP1 *is mutated, the overall expression level of all genes in the cluster is increased, and all 10 genes are now regulated (repressed) in the absence of N and/or L. Cluster #13 is another striking example of this type of regulation. In WT-sib plants, the 8 genes in cluster #13 are modestly induced by +N/-L, however in *bzip1-1 *mutant plants, those 8 genes tend to be strongly and consistently induced by N (*p *= 3.85 × 10^-6^, see Additional file 4). These observations highlight the point that *bZIP1 *might be involved in bypassing gene regulation (e.g., by N for cluster #13) needed in other circumstances, such as a different hydro-mineral environment, or at a different developmental stage.

The fourth class of genes (clusters #4, 17 and 20) show a complex de-regulation in response to N and/or L treatment. We hypothesize that these clusters might represent the composite effects of the three cases illustrated above. For example, cluster #20 contains genes clearly repressed by N in the dark (-L) in WT-sib, and induced by N in the dark in the *bzip1-1 *mutant. This partial reversion of the N effect (which occurs only in dark conditions) could correspond to the phenomenon described above.

To determine the biological significance of these gene clusters, we searched for the overrepresentation of MIPS functional terms among the genes on these clusters using the BioMaps tool from VirtualPlant [25]. This analysis did not uncover any significant terms. One very likely explanation for this negative result is that the number of genes in each cluster is too limited (≤ 20). As such, it is very unlikely to get any significant over-representation of terms in such small gene lists.

On a cluster-by-cluster basis, we developed conceptual Boolean models for illustrating the multiplicity of the roles of bZIP1 in the control of gene expression by L and/or N, based on the ANOVA output generated for each cluster (see Figure 3, 4 and Additional file 4, for details see Methods).

A striking result arises from the analysis of these Boolean models as a whole. Indeed, the underlying signaling mechanisms involving bZIP1 tend to use the same modality of expression. For example, when comparing clusters #9 *vs. *#10, it seems to be the exact same mode of regulation with opposite influences of the signal (L in this case) and of the *bZIP1 *mutation. This observation is also valid for more complex behaviors such as for clusters #13 vs. #14.

Taken together, these results demonstrate that bZIP1 is involved in fine-tuning gene expression in response to combined N and L signals. The role of bZIP1 can be divided into several modes of regulation, including the bypassing of N and L signals that can only be revealed when *bZIP1 *is absent.

## Discussion

### Identification of bZIP1 regulatory network(s)

Members of the bZIP transcription factor family are involved in the regulation of diverse biological processes such as plant growth, development, and environmental responses [16]. Several studies have reported that the transcript levels for the *bZIP1 *transcription factor are induced by dark treatments [7,10,12,26]. By over-expressing the bZIP1 protein in a protoplast system, it was shown that bZIP1 and other bZIP transcription factors act synergistically with the kinase (KIN10) in the control of dark-induced genes, including ASN1/DIN6, involved in asparagine synthesis [12]. In another study related to N-sensing, *bZIP1 *mRNA was also shown to be induced by N-treatment and, based on network analysis, it was predicted to control the N-induction of a set of predicted downstream target genes including ASN1 [10]. Herein, we assessed the complexity of the underlying regulatory networks controlled by bZIP1 (directly or indirectly) by studying the effect of the bZIP1 mutation in altering the L and/or N regulation of genes at a genome-wide level. To this aim, we used a T-DNA insertion line in the unique exon of the *bZIP1 *gene, and compared its expression pattern to a wild-type sibling with no T-DNA insert in bZIP1 gene (see Methods for more details). It is important to clarify that in this work we compared T-DNA insert and WT siblings in order to subtract the effects of potential T-DNA insertions. In order to rule out the possibility of the effect of a genetically linked insertion that could not be accounted by the WT siblings, we looked at the expression of the neighbour genes of bZIP1. First, the genes framing bZIP1 (At5g49440 and At5g49460) are normally expressed (signal > 200 and > 1000 respectively) and not affected by the T-DNA insertion. Second, since bZIP1 is in the 5^th ^chromosome, we evaluated how the mutation might have affected gene expression on this chromosome. We compared the expression of the genes in WT-sib vs. *bzip1-1 *mutant over the 5^th ^chromosome. Except for bZIP1 itself, which of course record a 32 fold repression of the bZIP1 transcript, we did not recorded any change in gene expression higher than a 4 fold down regulation in the bZIP1 mutant for a gene being on the 5^th ^chromosome. Moreover, this down regulation concerns a gene that is reported as not expressed according to the Affymetrix MAS5 calls and is ~1330 genes away from bZIP1. So we believe that the effect recorded for the bZIP1 mutation is very unlikely explainable by an extra T-DNA insertion that could be genetically linked to the actual insertion in bZIP1 genes.

The ANOVA analysis of these experiments uncovered a set of 219 genes whose regulation by N and/or L was altered in the *bzip1-1 *mutant, compared to a wild-type sibling, the results of which are discussed below. Interestingly, we were not able to detect mis-regulation of *ASN1 *gene expression in this transcriptome data set, even though ASN1 had previously been shown to be a target of bZIP1 in gain of function experiments related to dark regulation [12], and it had also been predicted to play a role in N-regulation [10]. This is likely due to the potential functional redundancy of the bZIP transcription factors, as cited by Baena-Gonzalez et al. [12], whose analysis shows that there is a redundancy with other bZIP genes in the control of ASN1/DIN6.

Another line of evidence supporting the complex role of bZIP1 in integrating N and L signals is the comparative transcriptome behavior of gene clusters in *bzip1-1 *mutant vs. a WT-sibling. Indeed, these results demonstrate that the bZIP1 mutation triggers a genome-wide deregulation in response to N and/or L signals that range from i) a lowering of the L signal effect, to ii) unlocking gene regulation in response to N and L combinations (discussed below). This provides evidence that the regulatory networks involved in the co-control of gene expression by N and L is highly complex and might involve several layers of regulations including functional redundancy. However, it is noteworthy that we were not able to find any positive evidence for transcriptional compensation by other bZIP genes (e.g., up-regulation of other bZIP genes in the *bzip1 *mutant).

Recently, the same *bzip1 *T-DNA mutant (SALK_059343) [23] was used to study effects of bZIP1 in carbon signaling by comparing the bZIP1 mutant and WT plants in a microarray analysis. In that study, RNA gel blot analysis confirmed that the SALK_059343 line was indeed a bZIP1-KO. As a result of that study, researchers found two sets of putative bZIP1-regulated genes. Among them, sugar-responsive genes are highly over-represented, implicating a role of bZIP1 in sugar-mediated gene expression. That work supports our present results indicating that a knock-out of the bZIP1 transcription factor is enough to drive dramatic changes in gene expression in a direct and/or indirect way.

### A new insight into the regulatory network complexity: hidden/locked regulatory mechanisms?

The role of transcription factors in integrating plant responses to nutritional cues is of great interest in order to shape and improve plant development in response to environmental nutrition. However, while several studies have studied genome-wide responses of mutants in response to a single nutrient treatment such a nitrate [27], to our knowledge only one [5] has reported the changes in gene regulation triggered by a mutation (*cli186*) in response to combinatorial treatments of signals (in that case, carbon (C) and light (L). In the present study, we have used a similar complete set of combinatorial treatments of L and N on the *bzip1-1 *mutant, to explore the role of bZIP1 in the interaction and propagation of these signals. Our combination of statistical analysis (ANOVA), clustering and Boolean modeling of the signals, allowed us to propose that a mutation in bZIP1 triggers different modes of de-regulation in response to N and/or L signaling. Previous work had already shown that transcription factors can be at the same time inducers and repressors, and our results on bZIP1 supported this hypothesis. However, we believe that our study went a step further. Indeed, the surprising results are that a mutation in bZIP1 can: i) invert the regulation of certain clusters/genes in response to nutritional signals (e.g., cluster #15 and 16), and ii) lead to the regulation of genes by N and/or L, which are not normally regulated in the WT context. Despite our extensive effort to find such events in the literature, we did not find any similar mechanism demonstrated at a genome-wide scale. This may be due to the fact that studies involving modeling in combined experimental treatment conditions (e.g. two signals) of wild-type and mutants are relatively rare. Interestingly, discovering the underlying mechanisms by which the mutation of a transcription factor leads to unlock and/or reverse regulation of genes by signals could have potential applications in biotechnology, since the use of mutants could potentially avoid the use of transgenesis in order to drive the regulation of target genes of interests in crop plants.

Finally, in order to assign biological functional categories significantly regulated to the gene lists under the control of the different signals nitrogen (N), light (L), genotype (G) and their interaction, we used the BioMap tool provided thought the Virtual Plant platform [25] (see Additional file 5). Surprisingly, we found several functional categories regulated as a group by N, but none of the category such as *nitrate assimilation*. This was an intriguing result. However, with the antecedents of the mis-regulations found by previous studies, we hypothesized that this could be due to the fact that some genes tend to be N regulated in the bZIP1 mutant, and not in WT. Thus, because genes regulated by N are detected as such by ANOVA over the WT and bZIP1 mutant data, we decided to analyze the same data but only on WT (2 way ANOVA, L and N). Interestingly, *nitrate assimilation *and *nitrate metabolic process *are found to be functional categories over-represented in the N regulated genes in WT. This means that the functional categories found to be regulated by N in the whole dataset, are largely due to the contribution of both the WT *and *the bZIP1 N regulated genes (see Additional file 5). Further, it is noteworthy that genes under the control of bZIP1, as a factor (G, genotype) or in combination with N or L, did not share any significant functional categories. Despite the fact that this is a negative result, it can be explained by the fact that bZIP1 triggered very diverse mis-regulations as it was demonstrated in the Boolean modeling process.

## Conclusions

In this work, we have taken a reverse genetic approach combined with a statistical modeling of the transcriptome, to study the role of *bZIP1 *in mediating the integration of N and L signaling. We were able to show not only that bZIP1 mutation affect N and L regulation, but also that this regulation can have different patterns/modes of regulation. We believe that in addition to elucidating the role of *bZIP1 *mutation in the whole plant, we present a valuable pipeline of analysis that can help to define the role of different genes in a system view.

## Methods

### Plant Material

*Arabidopsis thaliana *(var. Columbia Col-0) was used as parental line. The mutant plant with a knock-out in the *bZIP1 *gene, named *bzip1*-*1*, contains a T-DNA insertion in the unique exon of the gene (At5g49450) (Figure 2A). The mutant was obtained from the T-DNA Collection at the Salk Institute (SALK_059343). Note that for comparative analysis a sibling with no T-DNA insert in bZIP1 gene was used as the WT plant, named WT-sib (Figure 2B).

### Identification of homozygous *bzip1-1 *T-DNA mutants and wild-type siblings

Genomic DNA was isolated from leaves according to the manufacturer's protocol (Qiagen, Chatsworth, CA). The genotype was determined by PCR on genomic DNA using primers flanking the insertion point for wild-type plants. Homozygous mutants were identified by PCR genotyping, using the following gene specific primers: LP, 5-CGAACAACTTCTCCCACTTTC-3, and RP, 5-GCCATTTACATGCAAGGTACC-3, in combination with the T-DNA specific primer LBa1: 5-TGGTTCACGTAGTGGGCCATCG-3. These primers were used to identify the presence or absence (WT sibling) of the insert (see Figure 2A).

### Plant Growth Conditions

Arabidopsis seeds were placed for 2 days in the dark at 4°C to synchronize germination. Seeds were surface sterilized and then sowed into plates containing a sterile mesh over the agar surface to facilitate their transfer into the treatment plates. Plates contained basal MS salts (custom-made; GIBCO) with 0.5 mM KNO_3_, 3 mM sucrose and 0.8% BactoAgar at pH 5.7. After 14 days under long-day (16 h light: 8 h dark) conditions with light intensity of 50 μE.m^-2^.s^-1 ^and at 22°C, plants were transferred to new plates containing 20 mM KNO_3 _and 20 mM NH_4_NO_3 _(referred here as 1xN: concentrations in MS media) in the absence or presence of light for 2 h at the start of their light cycle. Control plants were transferred toward a fresh media complemented with KCl, also in the absence or presence of light as a mock treatment. Light vs. dark treatments were done by simply leaving the treatment plates in the light growth chamber, or by covering them with double aluminium foil (dark treatment) for two hours. After these treatments, whole seedlings were harvest and immediately frozen in liquid nitrogen.

### RNA isolation and RT-qPCR

RNA was isolated from roots and shoots with TRIzol reagent (Invitrogen Life Technologies, Carlsbad, CA). RNA was previously treated with DNAse following the manufacturer instructions (Invitrogen, Catalog number 18047019). cDNA synthesis from whole mRNA extractions was carried out according to kit manufacturer protocol (Invitrogen, Catalog number 11146-024). Real time quantitative PCR was carried out using LightCycle FastStart DNA MasterPLUS SYBR Green I kit (Cat. No. 03752186001) with a LightCycler both from Roche Diagnostics, Mannheim, Germany. The following primers were used for amplification and detection: At4g24550 (putative clathrin coat assembly protein): 5'-AGCATACACTGCGTGCAAAG-3' (forward primer) and 5'-TCGCCTGTGTCACATATCTC-3' (reverse primer); bZIP1: (At5g49450), 5'-CGCAAGTTATCAAACCGCG-3' (forward primer) and 5'-CCACAACTCAATTTCCACGG-3' (reverse primer). Thermal cycling was performed as follows: initial denaturation at 95°C for 15 min, followed by 45 cycles of denaturation at 95°C for 6 s, annealing at 60°C for 7 s, and extension at 72°C for 10 s. Standards were prepared with a 10-fold serial dilution of the original cDNA control sample and were run under the same PCR conditions used for the samples. The amount of *bZIP1 *mRNA levels was normalized according to the amount of At4g24550.

### Microarray experiments and analysis

cDNA synthesis, array hybridization to the Arabidopsis Genome ATH1 array, and normalization of the signal intensities were performed as previously described in [8]. Three replicates corresponding to independent experiments (biological replicates) were done for each treatment/genotype. All microarray data was processed with Microarray Analysis Suite 5.0 software (MASv5.0). MASv5.0 Affymetrix call (Absent, Marginal or Present) were used to keep only probes having at least a Marginal/Present call in one of the 24 hybridization. Such a procedure facilitated the elimination of transcripts with very low signals in both treatments (declared "absent"). Affymetrix microarray expression data has been deposited in the Gene Expression Omnibus (GEO) database http://www.ncbi.nlm.nih.gov/geo/. GEO accession: GSE21601. Over-represented functional categories in the lists compared to Arabidopsis ATH1 whole genome array were obtained according to the MIPS classification, using BioMaps tool from the VirtualPlant webpage (http://virtualplant.bio.nyu.edu and [25]). A binomial method was used for the analysis with a *p*-value cutoff of 0.05.

### Modeling of gene expression patterns using ANOVA

Statistical analysis was performed as follows. All data manipulations were performed in R http://www.r-project.org/. The data set, corresponding to 24 ATH1 chips times 22,810 probes, were analyzed by an ANOVA in order to pre-filter genes regulated by the considered Factors. *aov() *function has been used over the data set where the signal of a probe-set *i *is Pi ~ αN+βL+γG+∂L*N+χL*G+ηN*G+λN*L*G, were N is the effect of the nitrogen treatment, L is the effect of the Light treatment, G is the effect of the genotype (WT-sib or *bzip1-1*) and, L*N; L*G; N*G are the effect of their first order of interactions, and N*L*G is the effect of their second order of interactions. Greek letters are the coefficient of the ANOVA. Further, we determined probes having a significant call (ANOVA *p *< 0.01 cutoff, corresponding to FDR < 20% across the whole analysis) for each factor and for their first and 2^nd ^order of interaction.

### Gene Clustering Analysis

Clustering was performed using custom made R functions using adapted script with following characteristics: *heatmap *function, metric: Pearson correlation, aggregation method: average. The number of clusters has been determined according to the Figure of Merit (FOM) method [28]. The clusters have been generated using a K-mean method on R using the function *cutree *[29]. We developed Boolean models for each cluster to illustrate the role of bZIP1 based on the ANOVA analysis run on the considered cluster data. ANOVA models were fitted on all the genes belonging to the studied cluster keeping the biological replicates separated. The Boolean modeling was built as follows: when signals were found to be significant on their own (single factor effect: N, L or G-bZIP1), the signal was drawn to directly influence the cluster expression (as in cluster 1 in Figure 3); when the signals (including bZIP1 mutation effect, G) were found to be interacting, a Boolean gate was drawn to reflect the direction of the interactions displayed in the cluster. For instance, in cluster 2: the ANOVA analysis detected that i) N and L interacts and that ii) N and L interacts with the bZIP1 mutation. Thus, the analysis of the cluster expression demonstrated that in WT the genes are not regulated, and in *bzip1 *mutant these genes are over-expressed. So, in this case, the bZIP effector arrow is shown to be repressive. Finally, the model shows that in the absence of N *AND *L this target gene cluster is over-expressed. Thus, we have built an *AND*-gate to express this (see Figure 3 cluster #2).

## Authors' contributions

MO, GK, MK and GC designed the study. MO and SR performed the experiments and molecular analysis. GK performed statistical analysis. MO and GK wrote the paper. All authors read and approved the final version of the manuscript.

## Supplementary Material

Additional file 1 **Regulated gene lists in *bzip1-1 *and WT sibling**. Genes are sorted based on their regulation according to the ANOVA analysis (*p*val < 0.01 cutoff): N, L, G, N*L, N*G, L*G, N*L*G; where N = Nitrogen, L = Light, G = Genotype (*bzip1-1 *vs. WT sibling). Click here for file

Additional file 2 **219 Genes misregulated in *bzip1-1 *compared to a WT sibling**. Union of the lists where genotype has an effect (G, N*G, L*G, N*L*G) based on the ANOVA analysis (*p*val < 0.01 cutoff). N = Nitrogen, L = Light, G = Genotype (*bzip1-1 *vs. WT sibling). Click here for file

Additional file 3 **Histograms show ATH1 array signals for the 219 genes regulated by genotype (*bzip1-1 *vs. WT sibling)**. The array data are averages of three biological replicates. Error bars = SE. Click here for file

Additional file 4 **ANOVA output for each bZIP regulated gene cluster (for more details see Methods)**. Click here for file

Additional file 5 **Functional terms statistically over-represented in bZIP1 regulated clusters**. Frequencies are percent of genes that are classified in a given MIPS functional group. Observed frequency refers to genes in a specific set. Expected frequency refers to genes in the Arabidopsis ATH1 whole genome array. Groups are ranked by their *p*-values, which were determined by comparing the observed with the expected frequencies for that functional group using the Binomial method. Click here for file
