# Supplementary figures and images for: Modeling the global effect of the basic-leucine zipper transcription factor 1 (bZIP1) on nitrogen and light regulation in Arabidopsis
Source: BMC Syst Biol. 2010 Aug 12;4:111. doi: 10.1186/1752-0509-4-111 (PMC2933594; doi:10.1186/1752-0509-4-111)

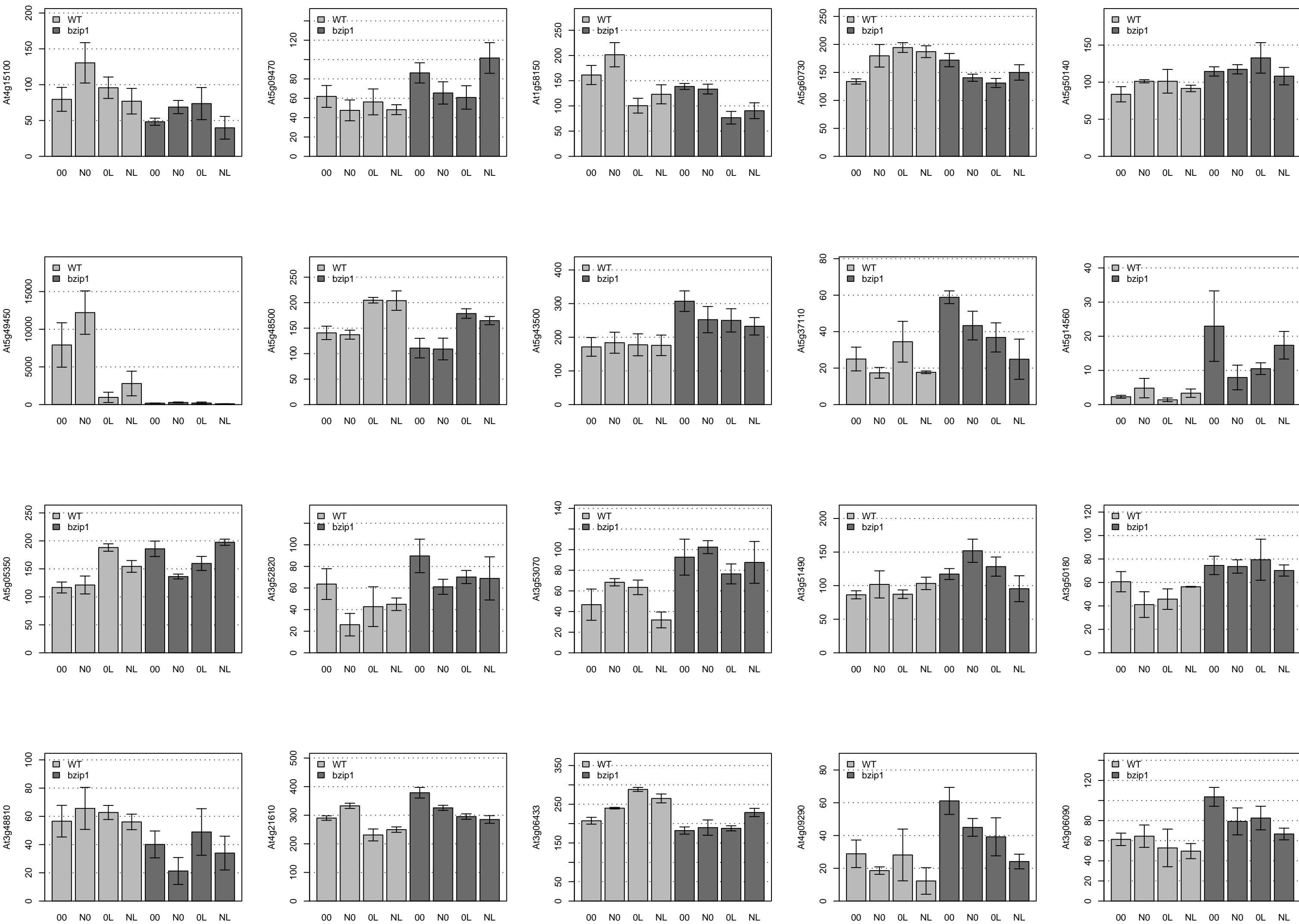

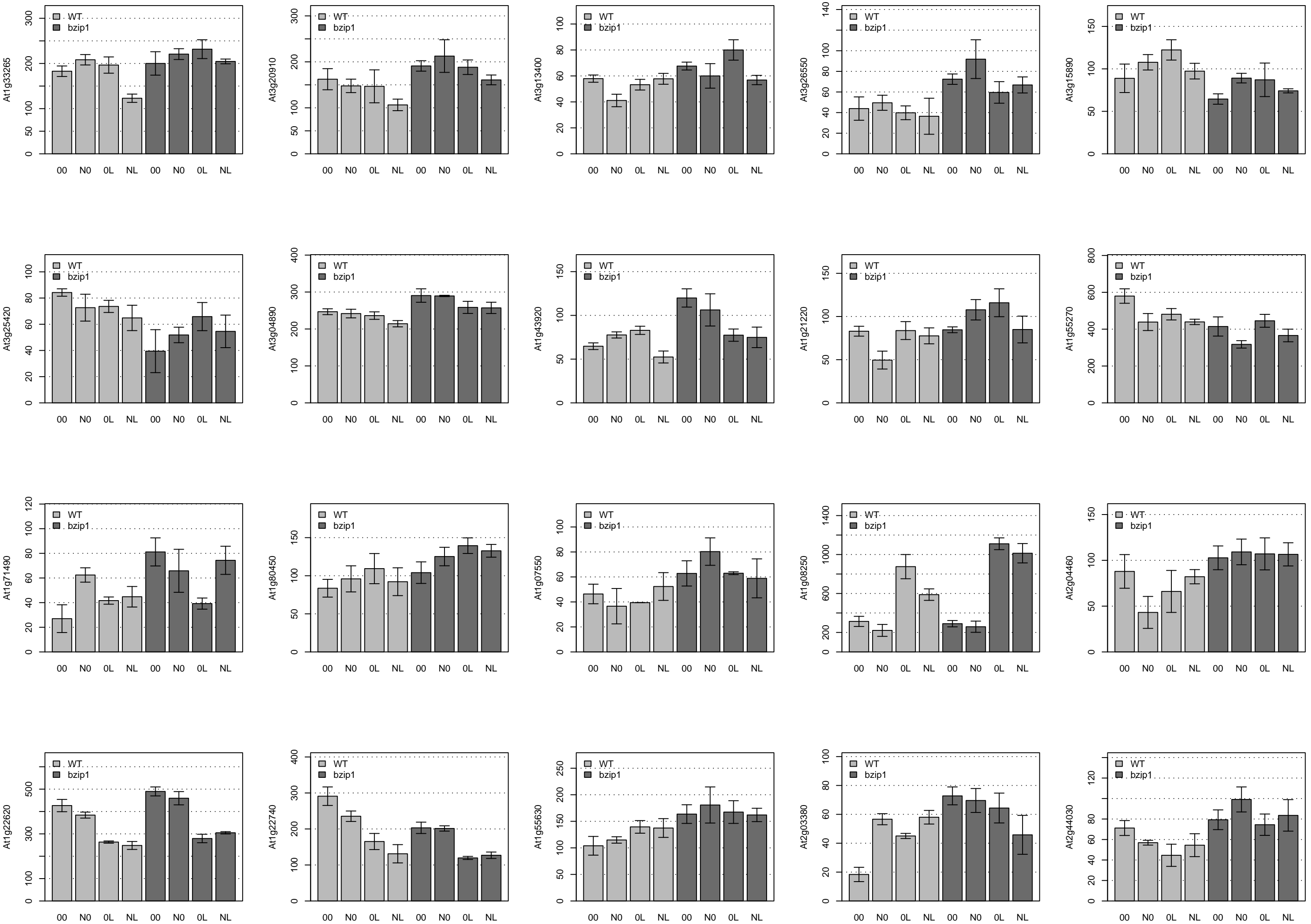

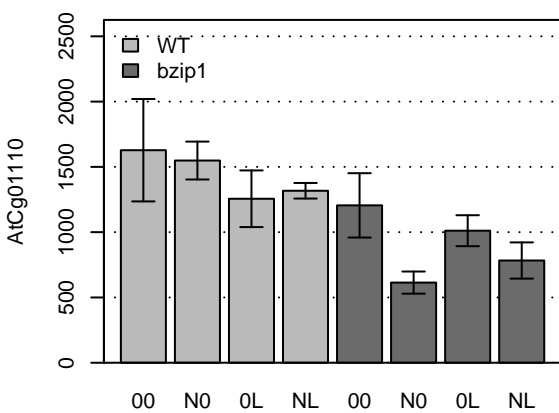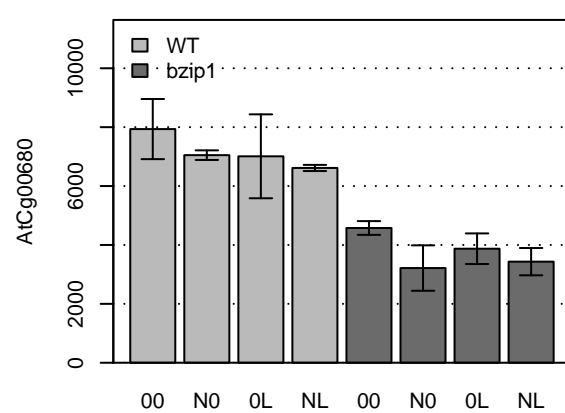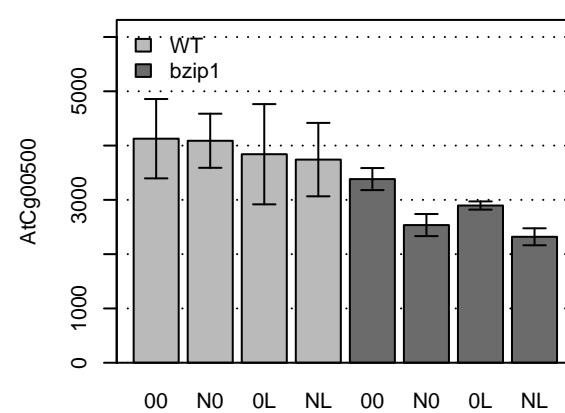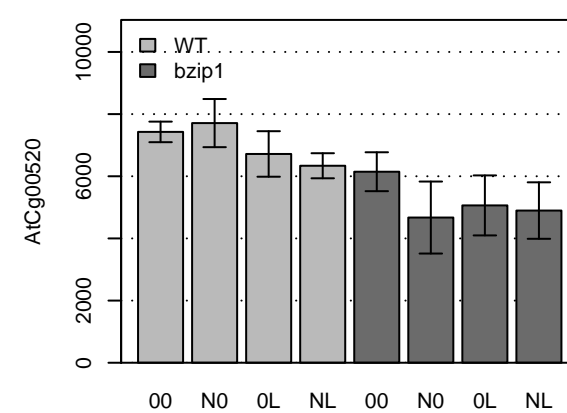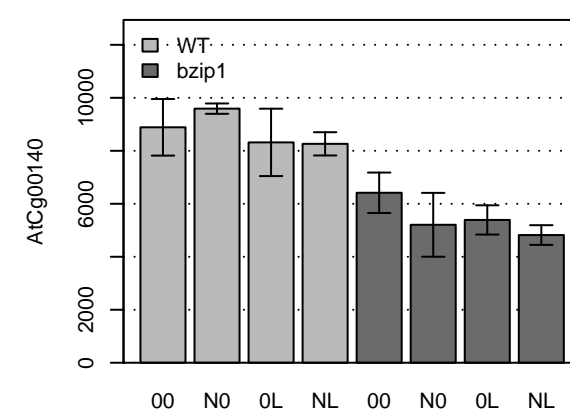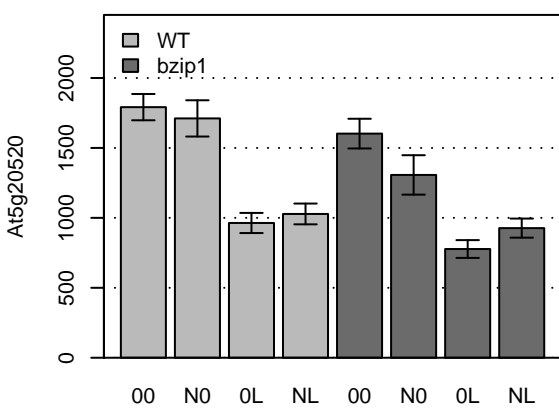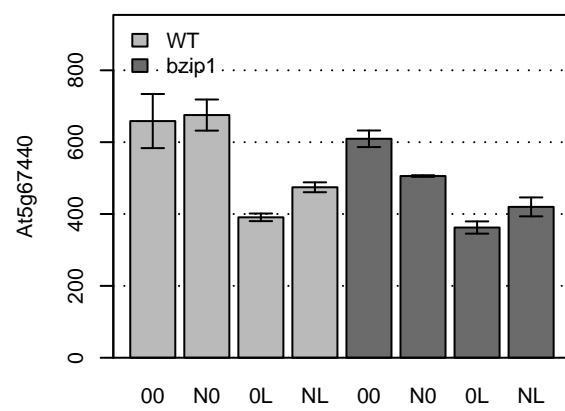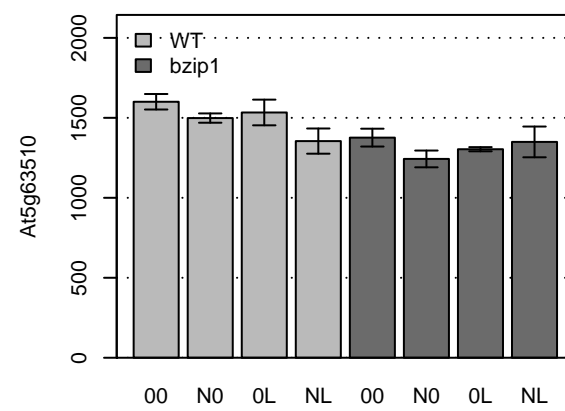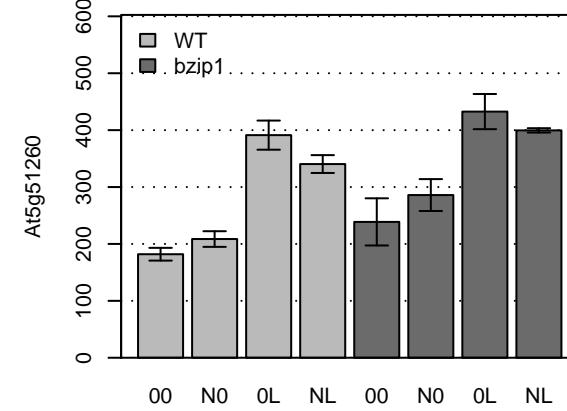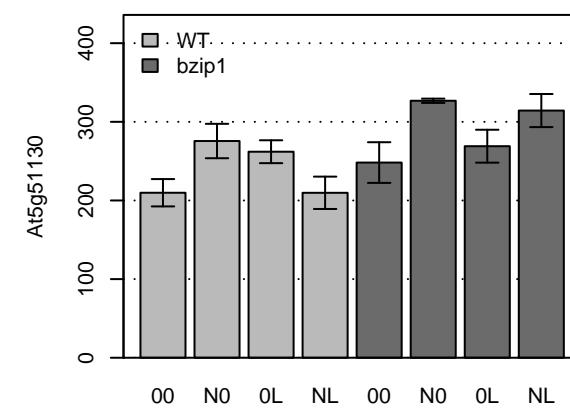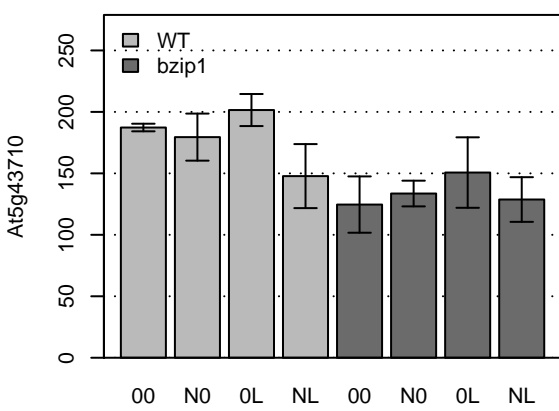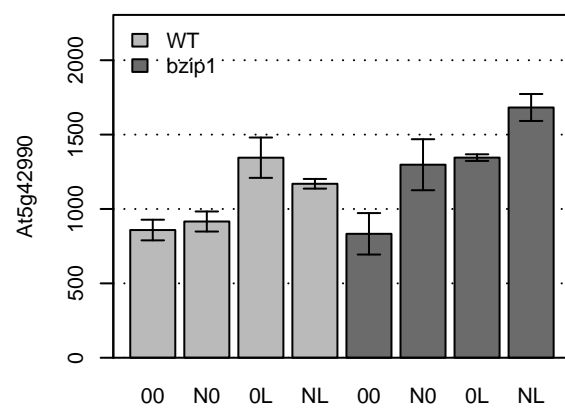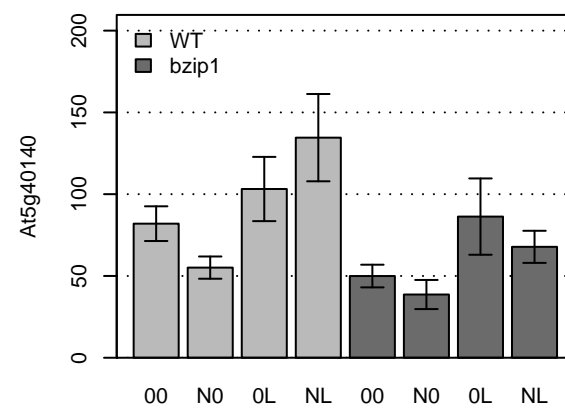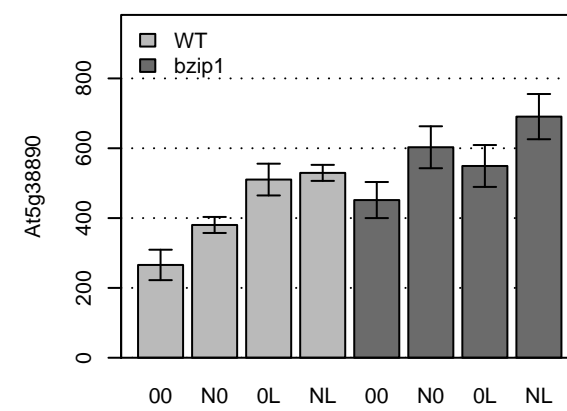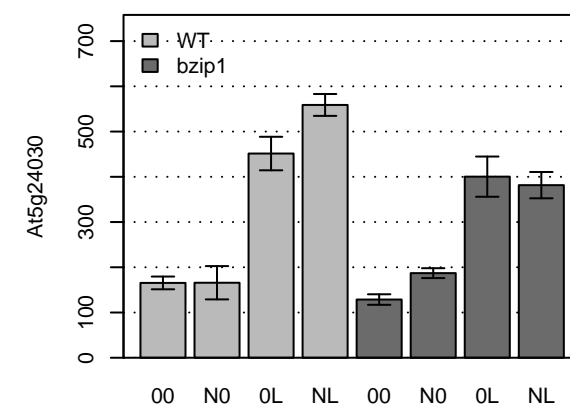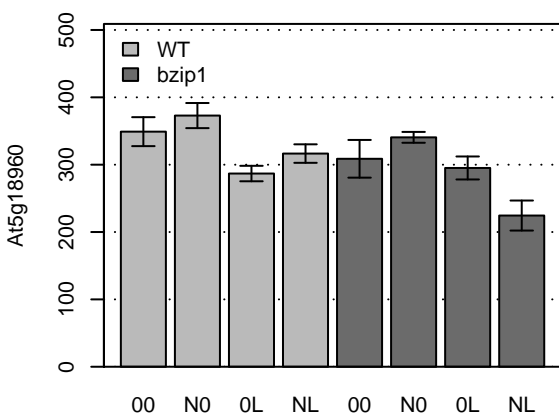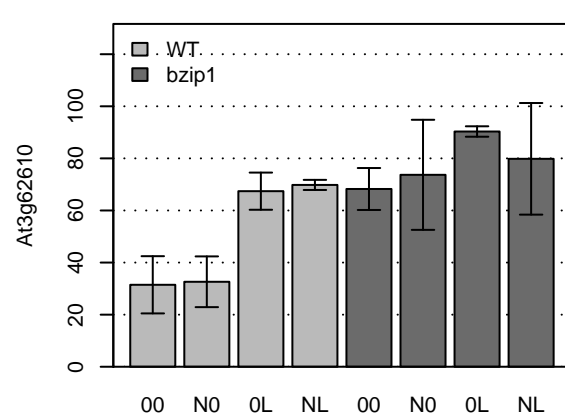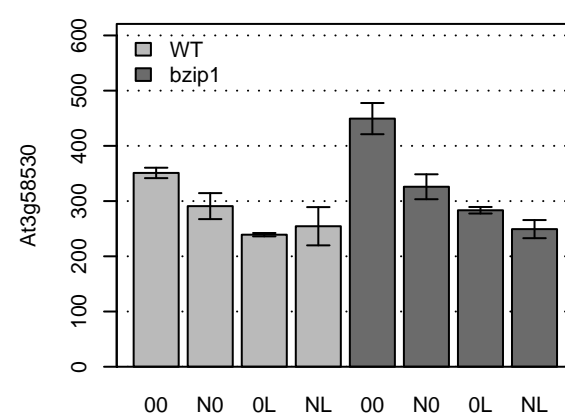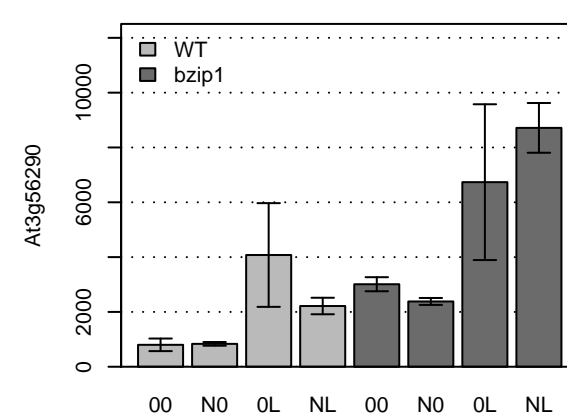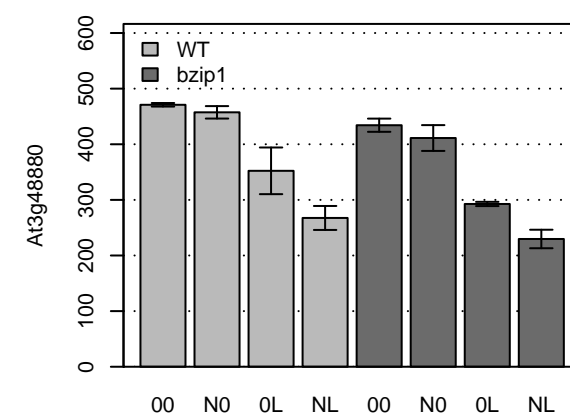

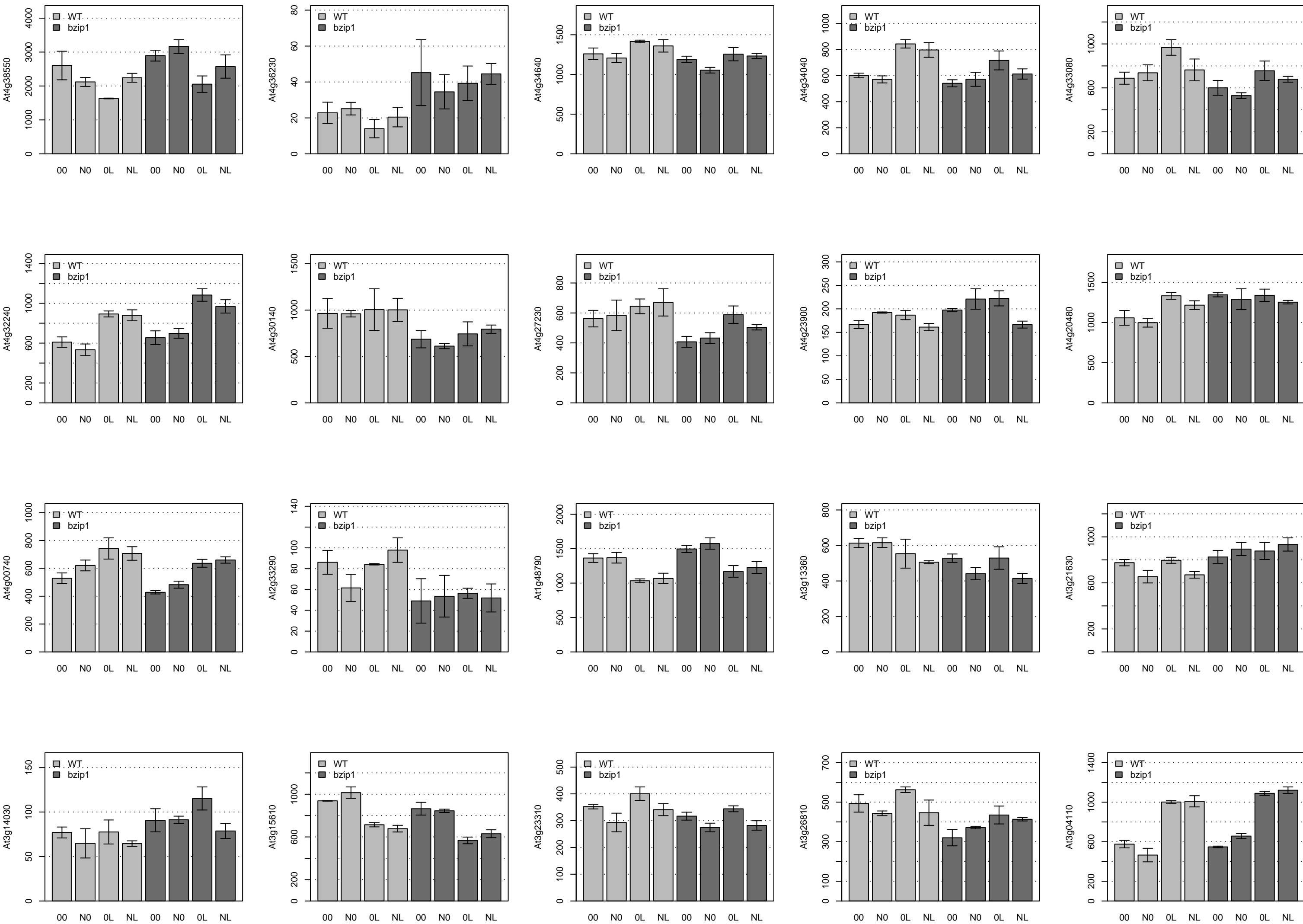

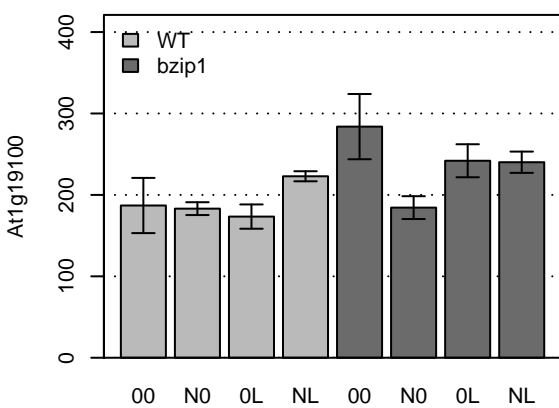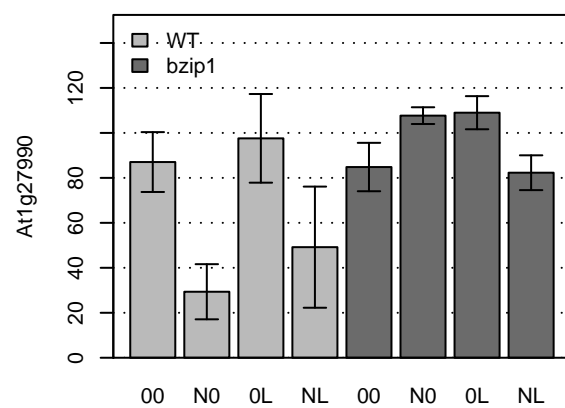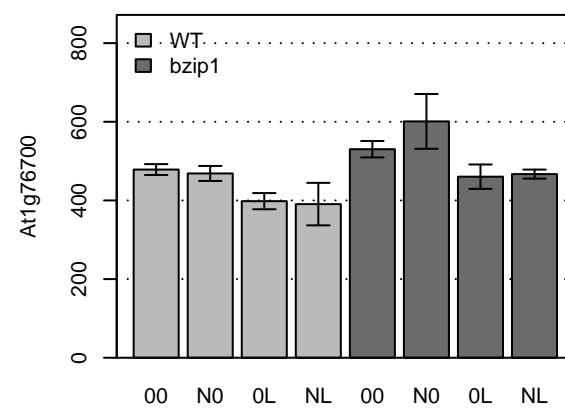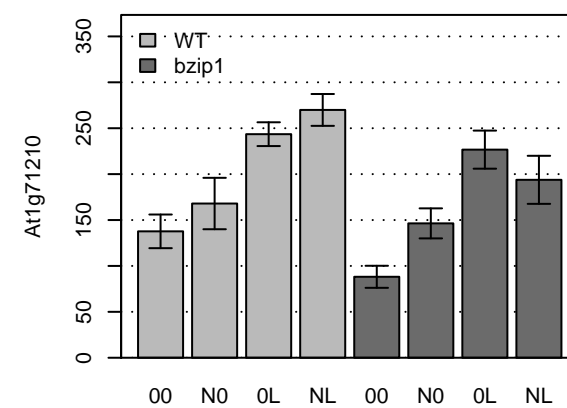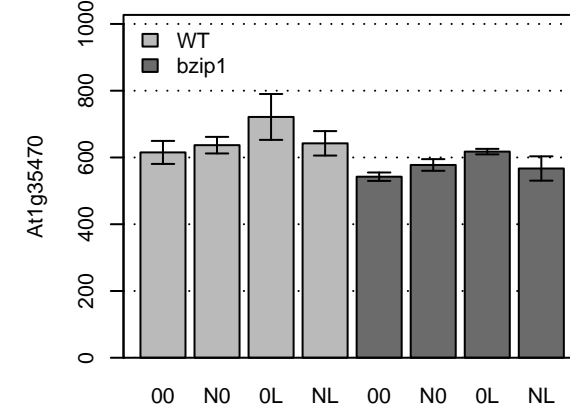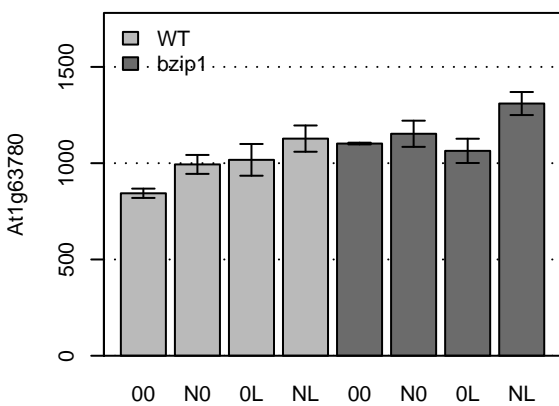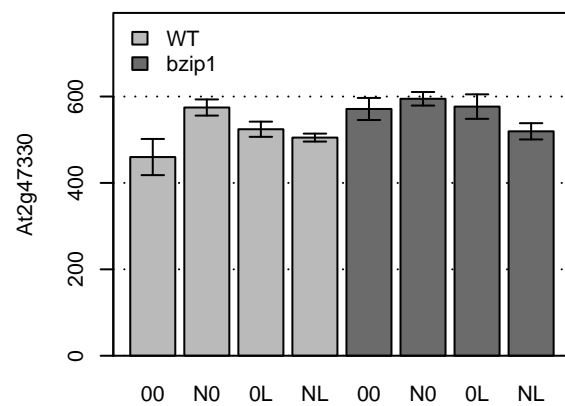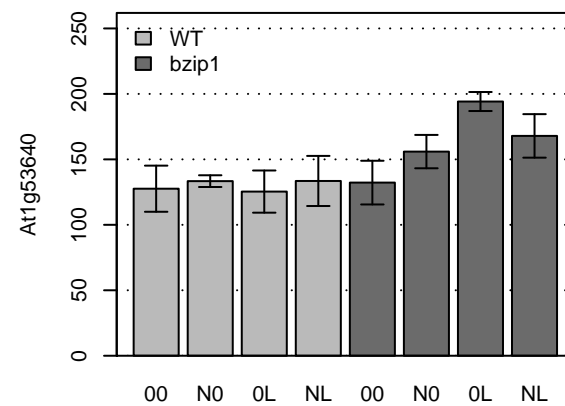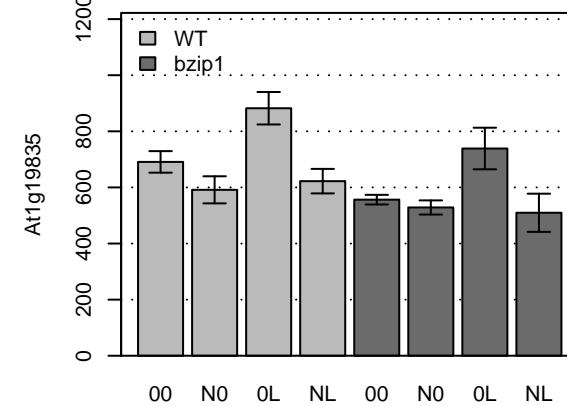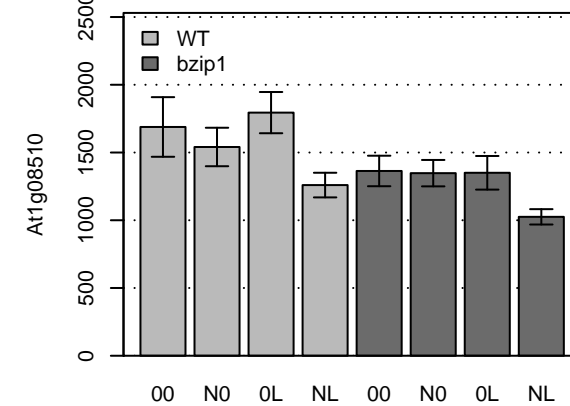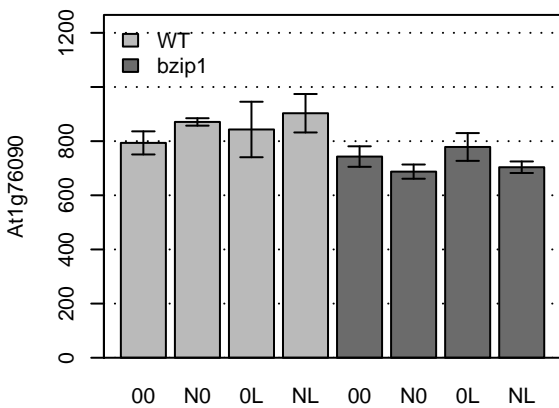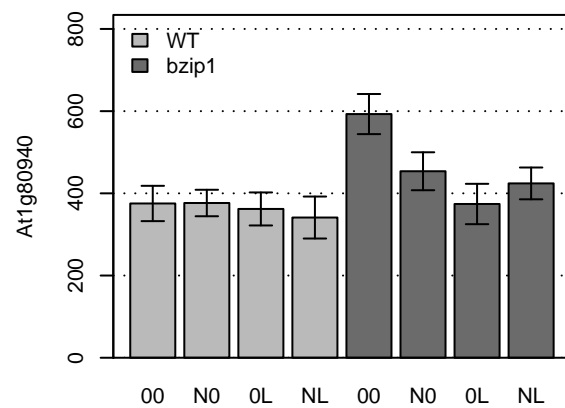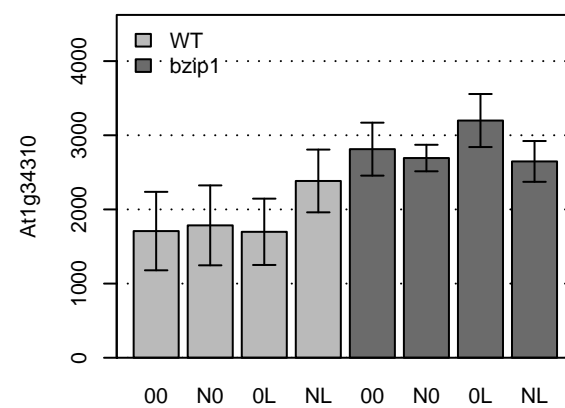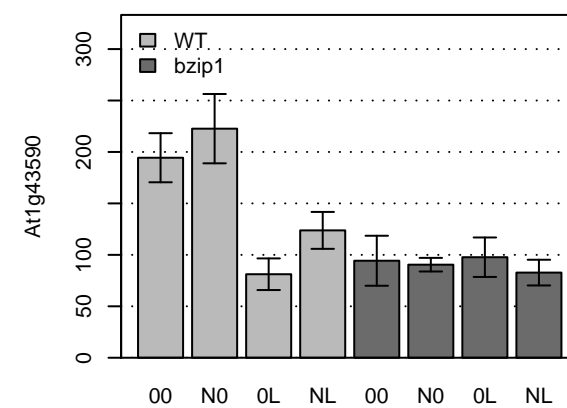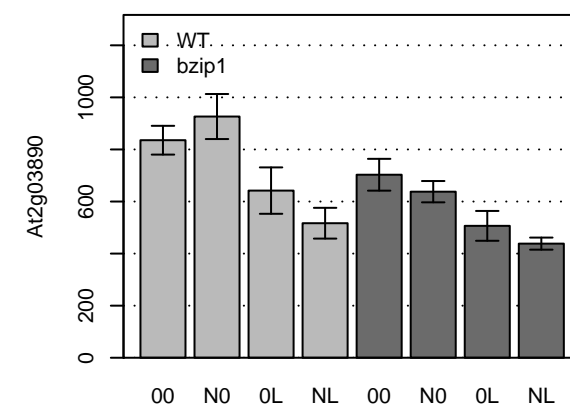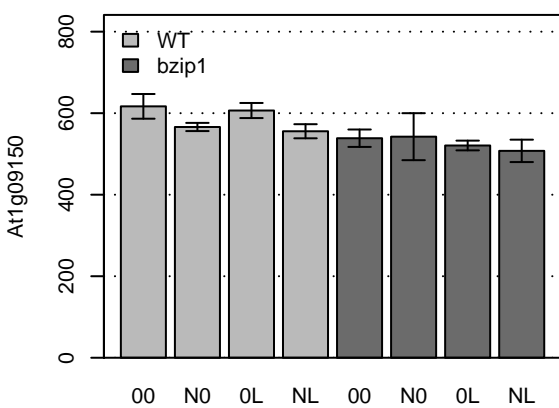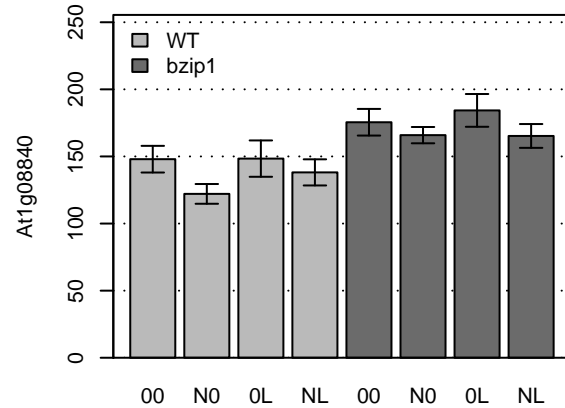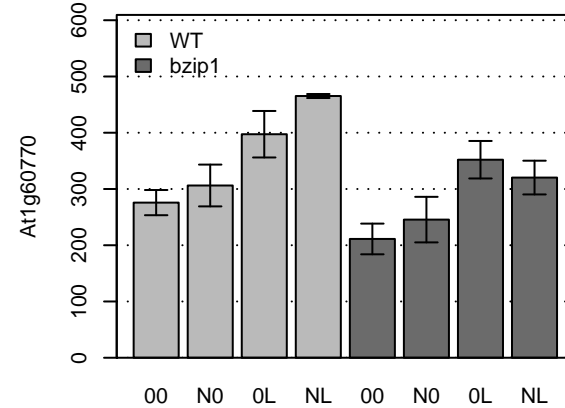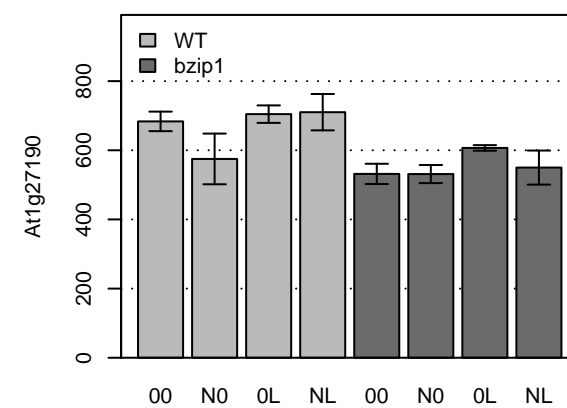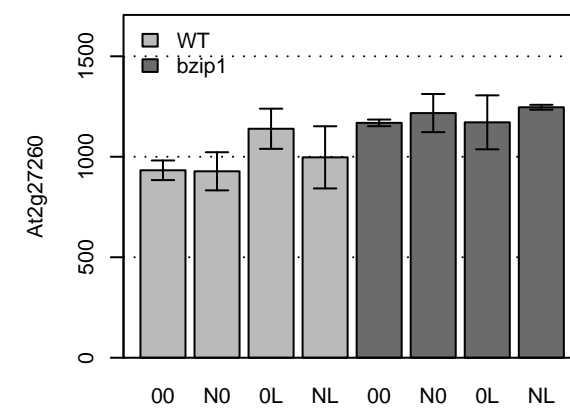

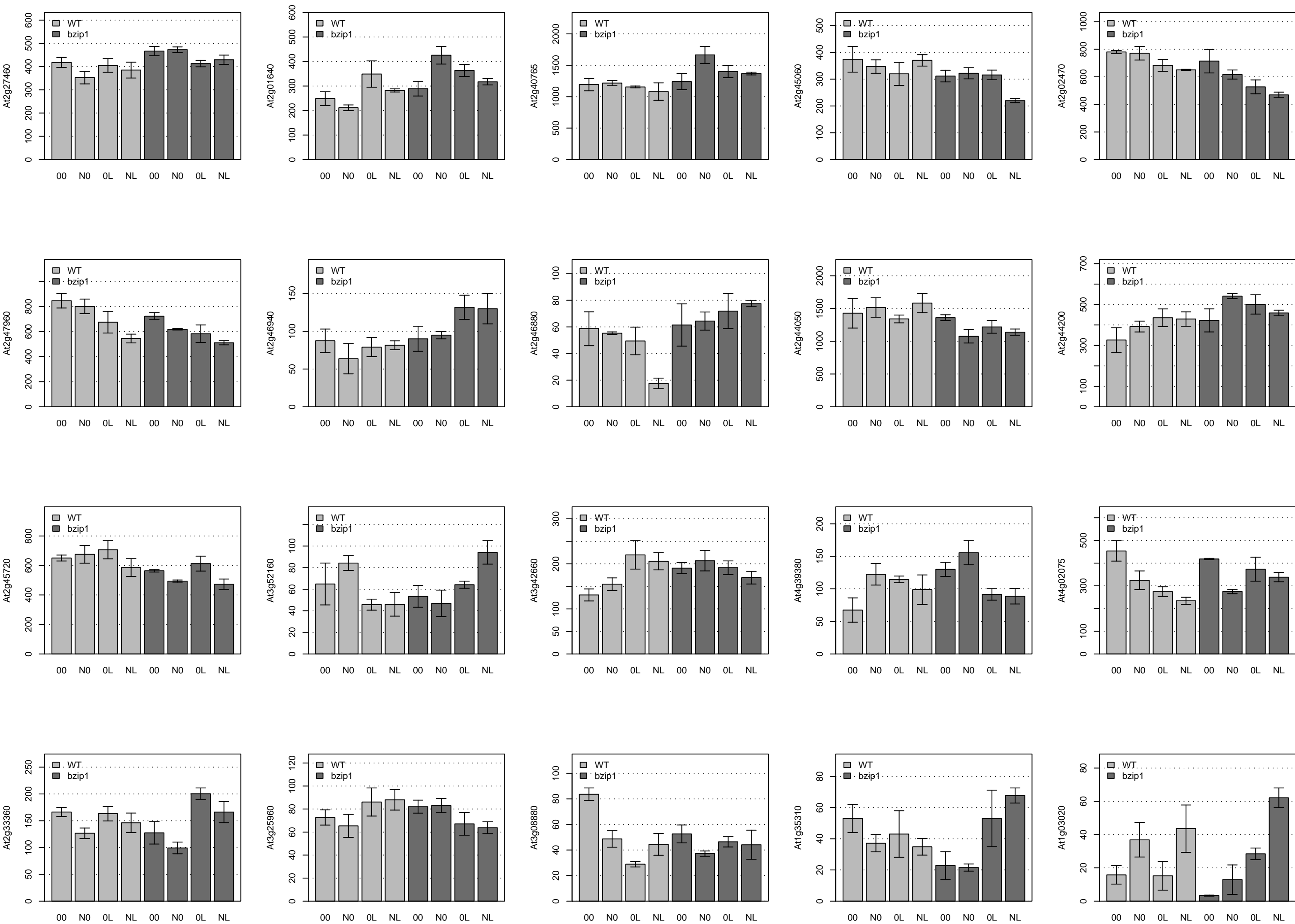

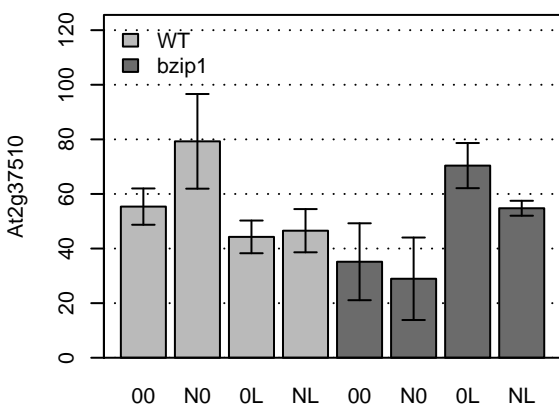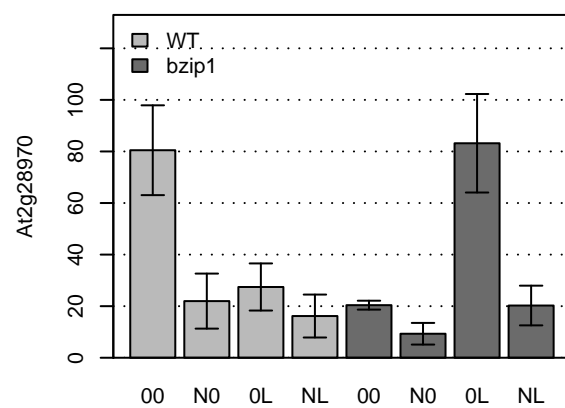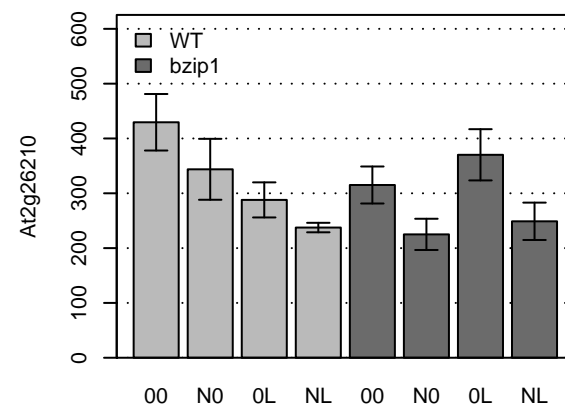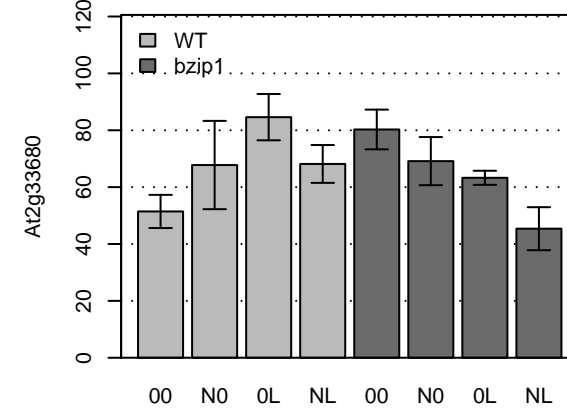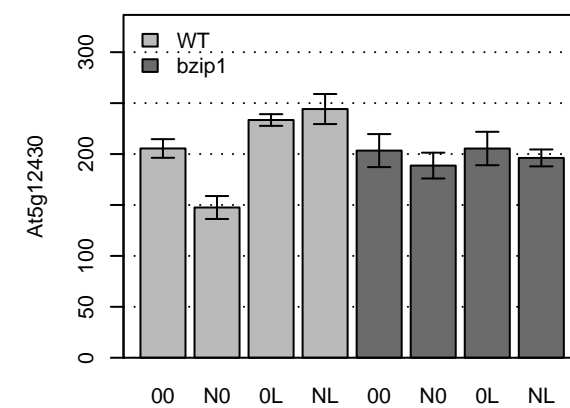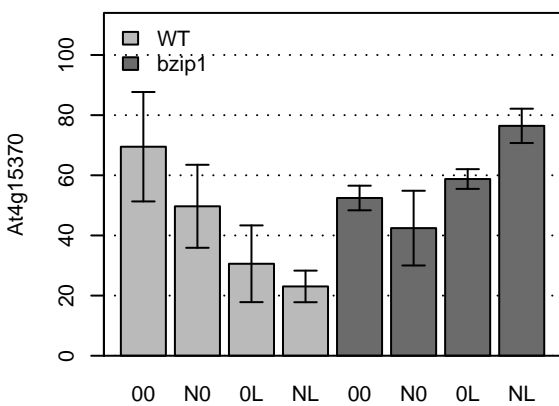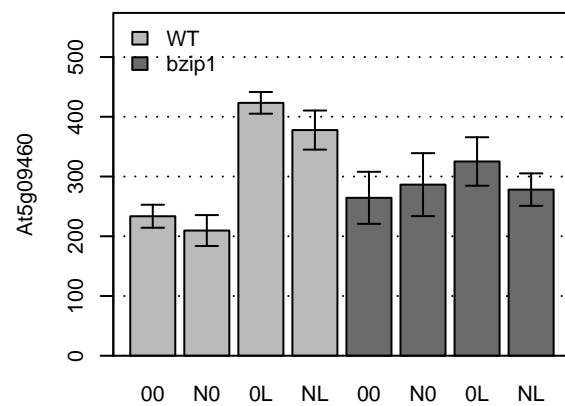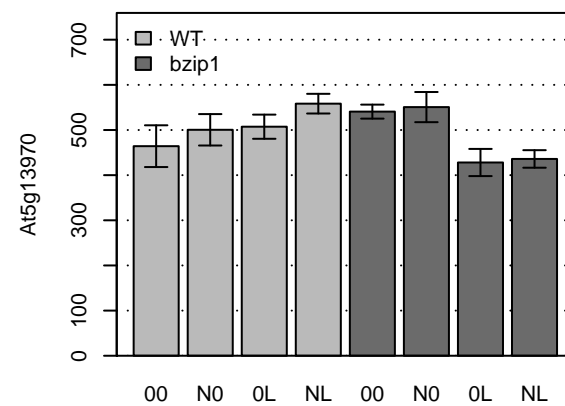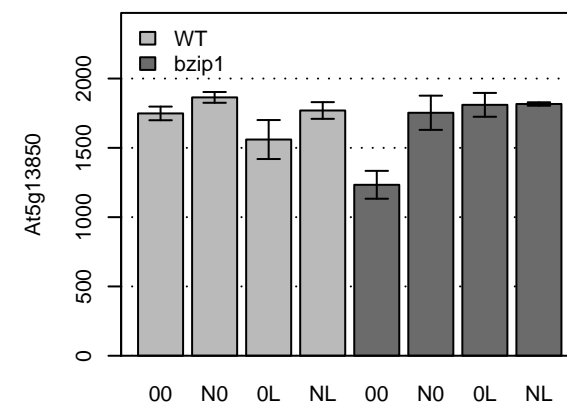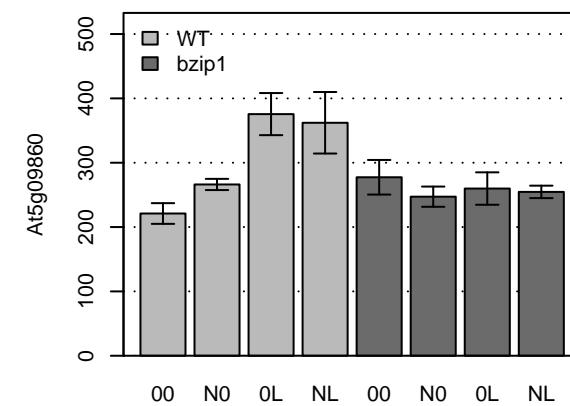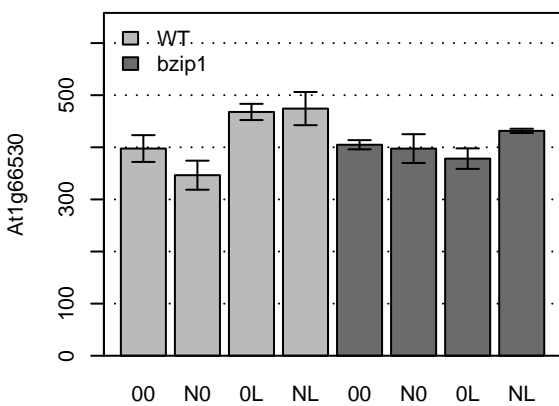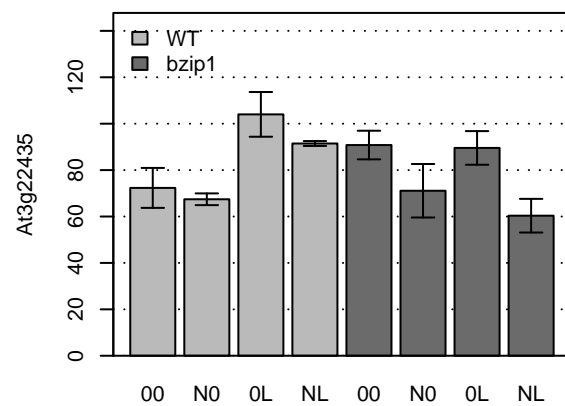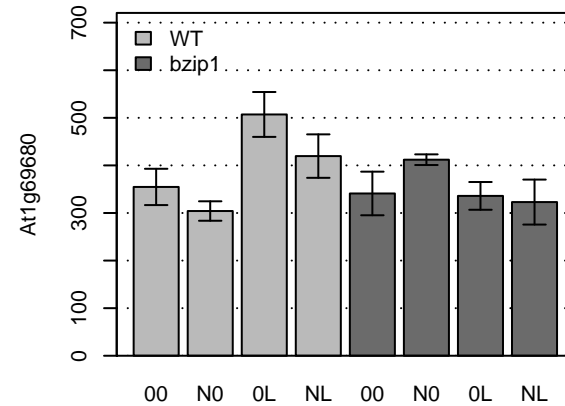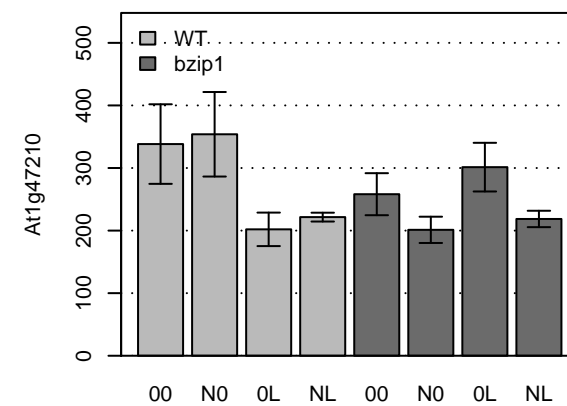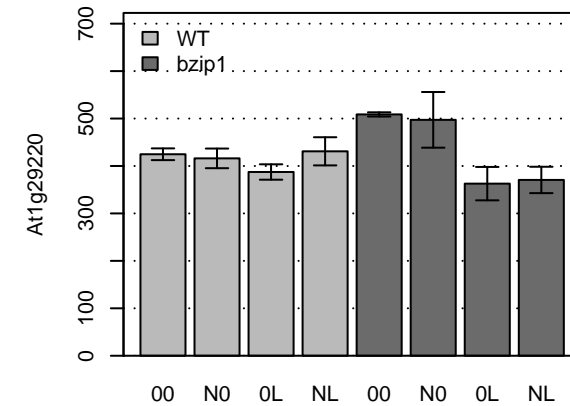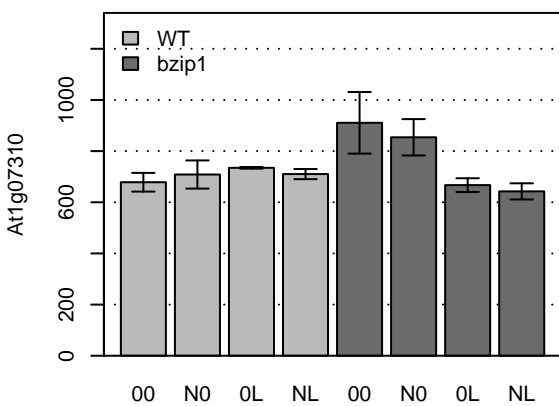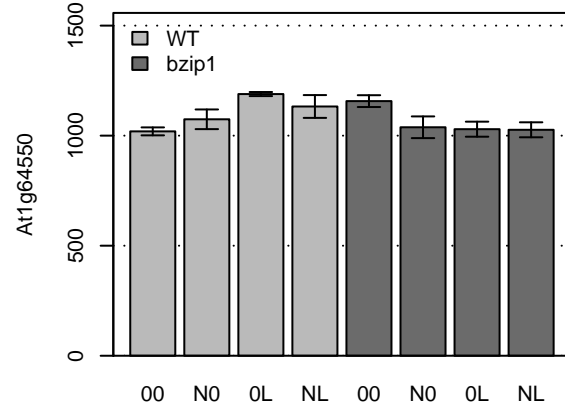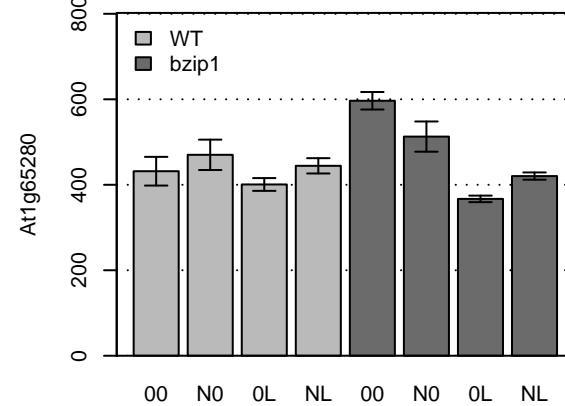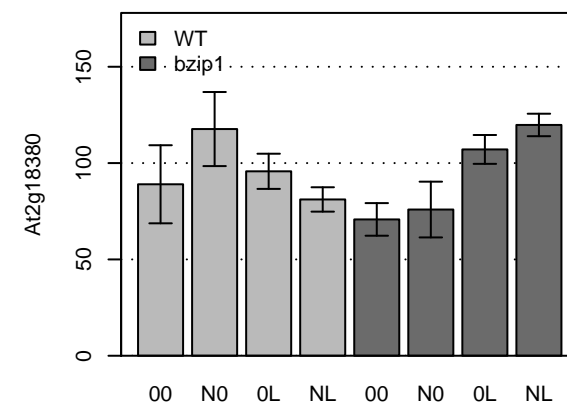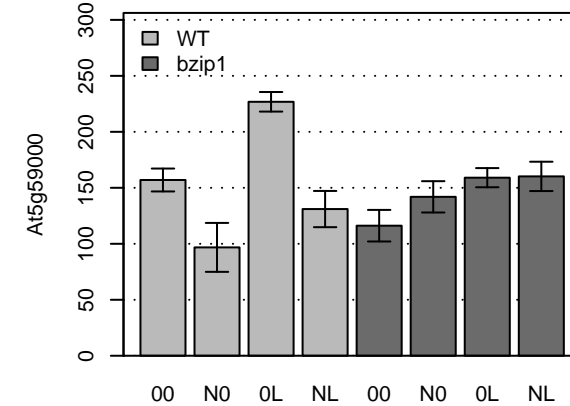

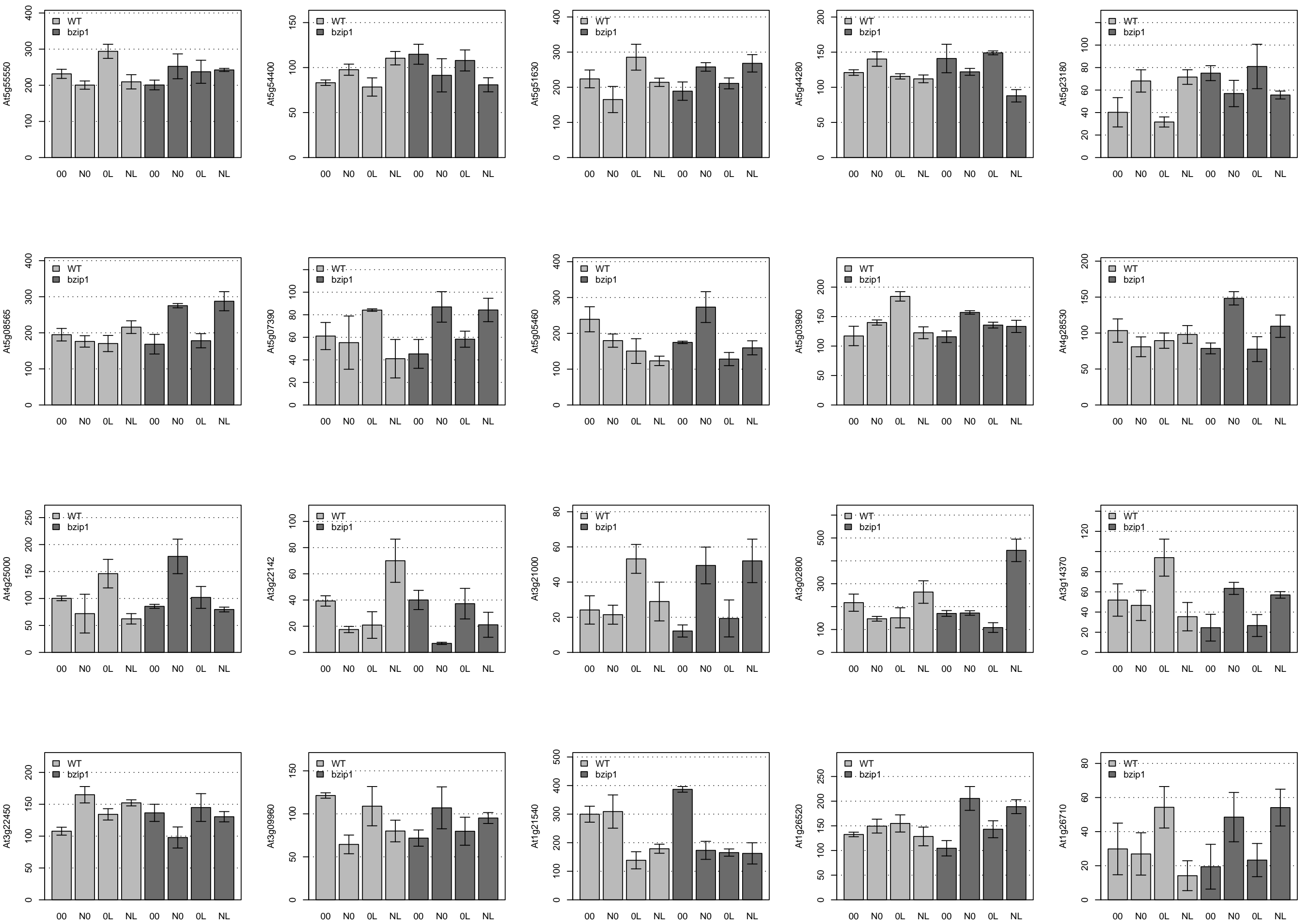

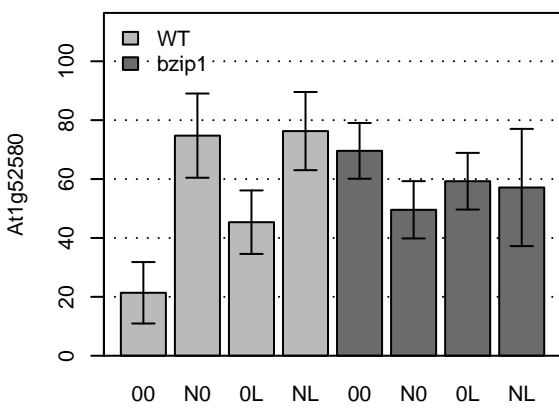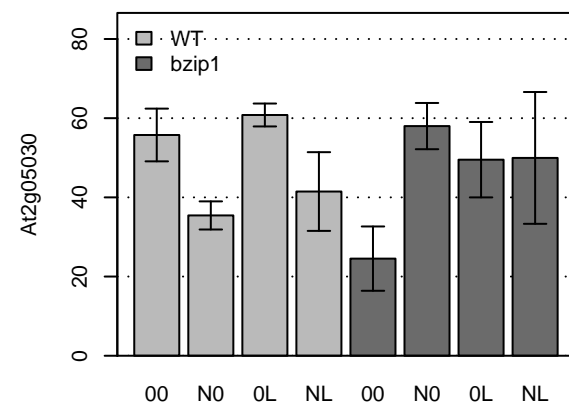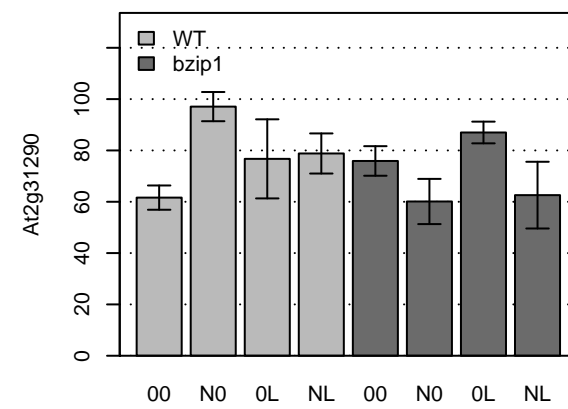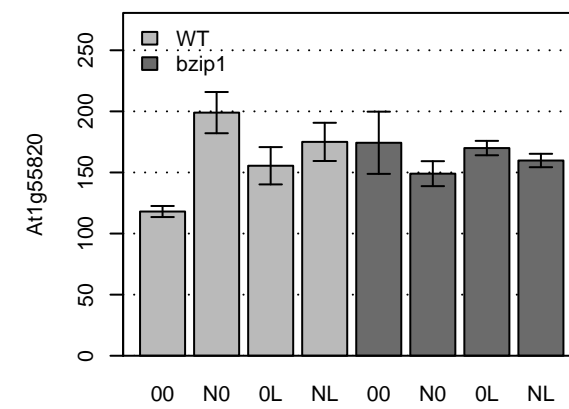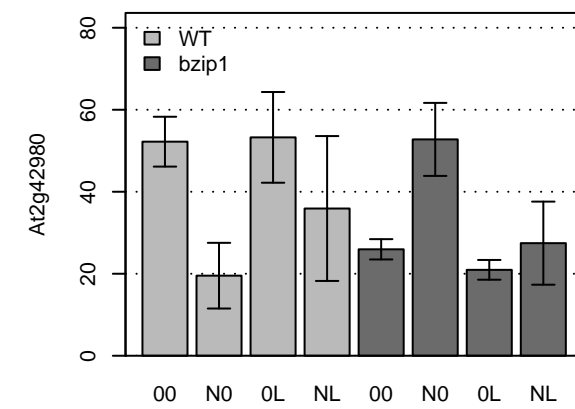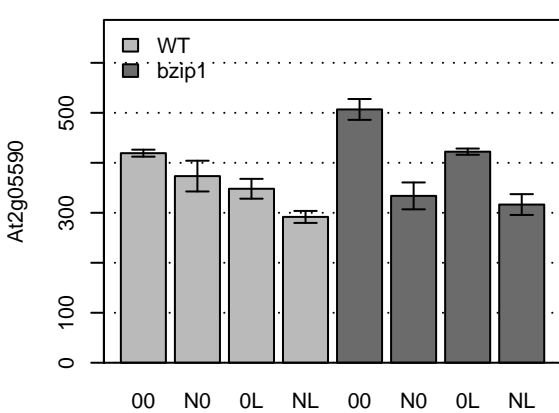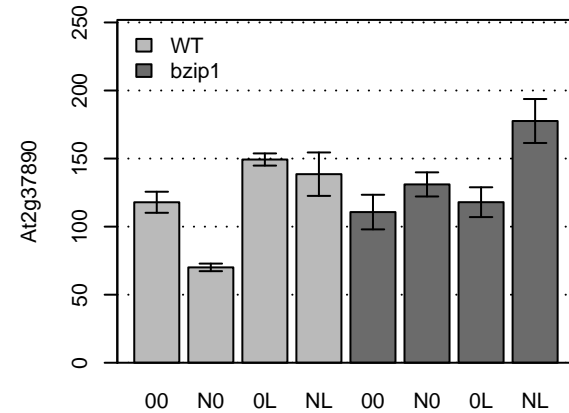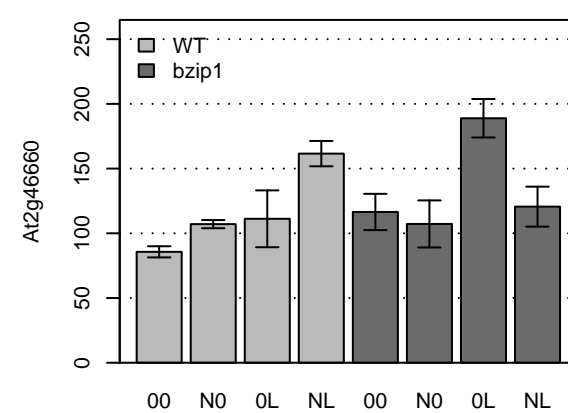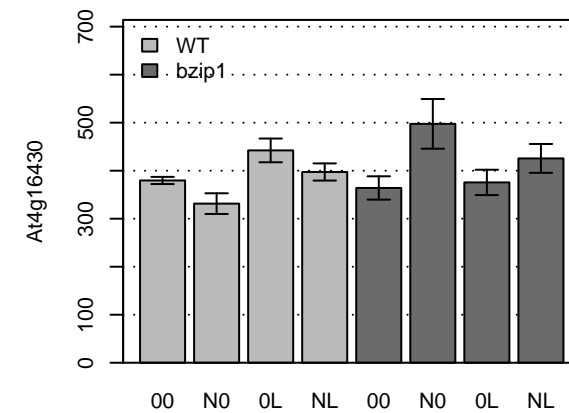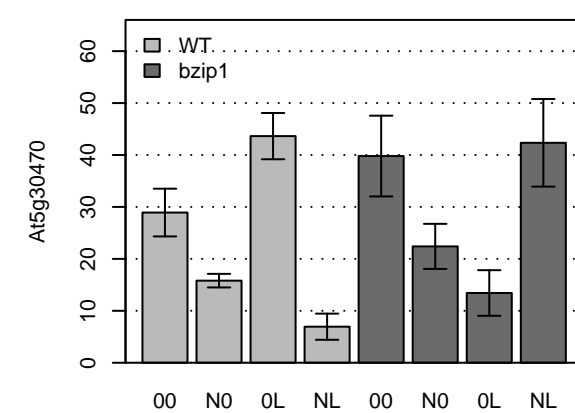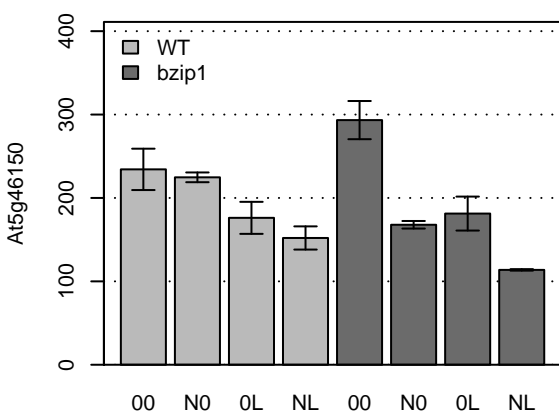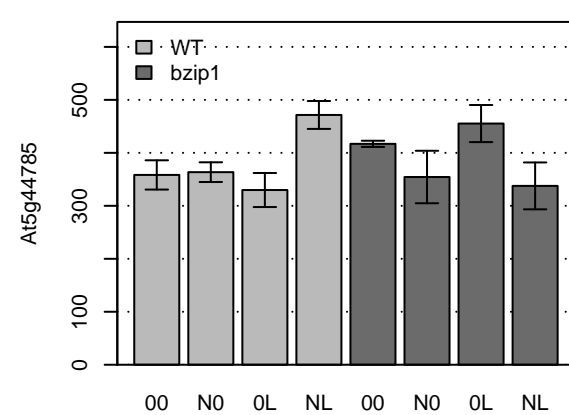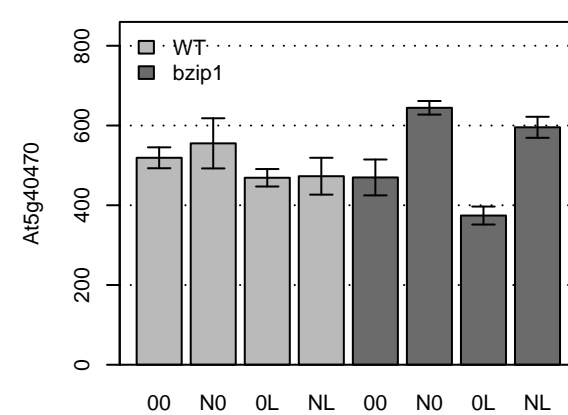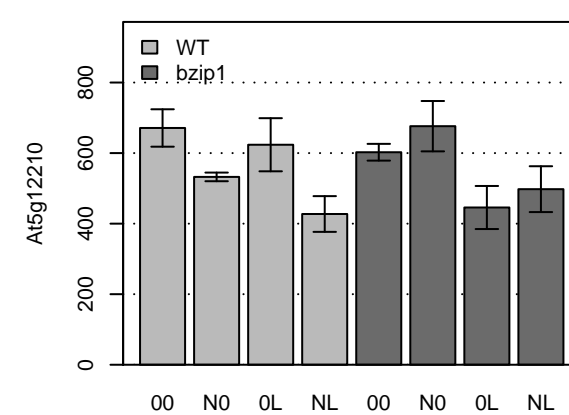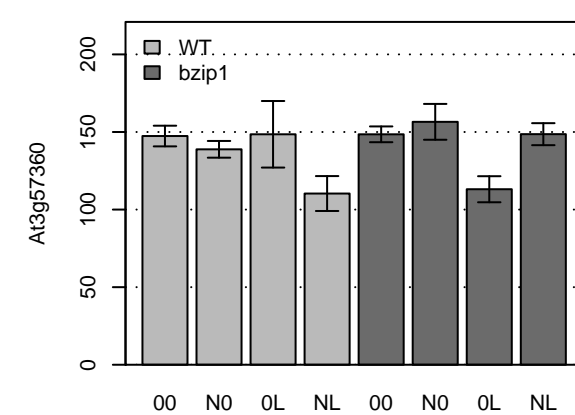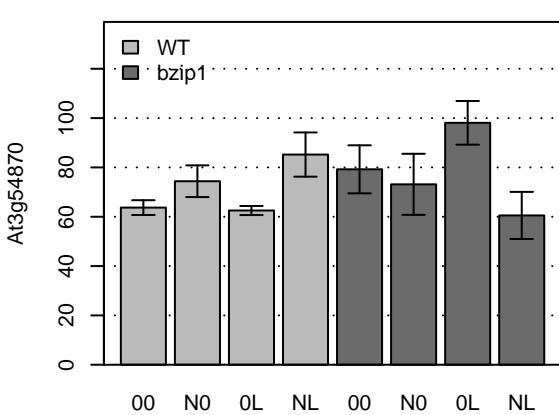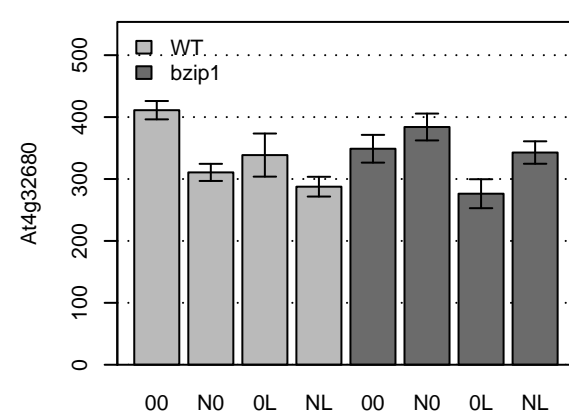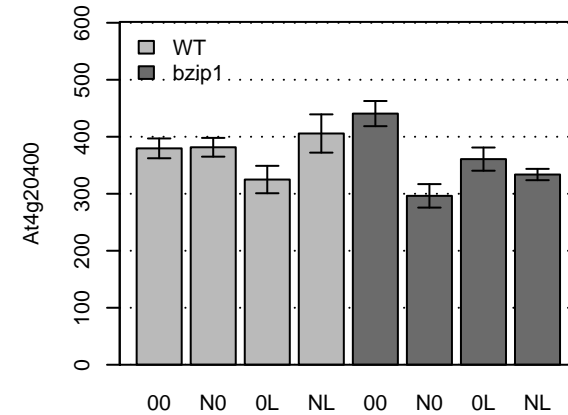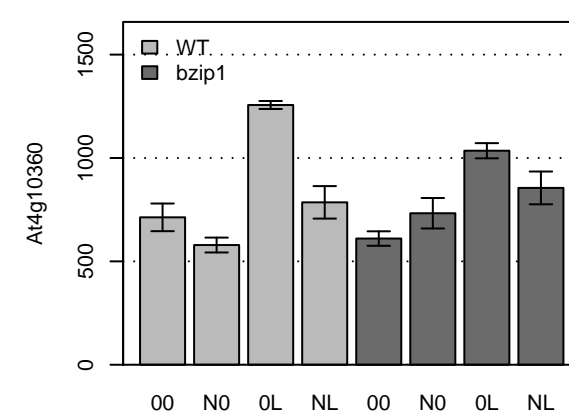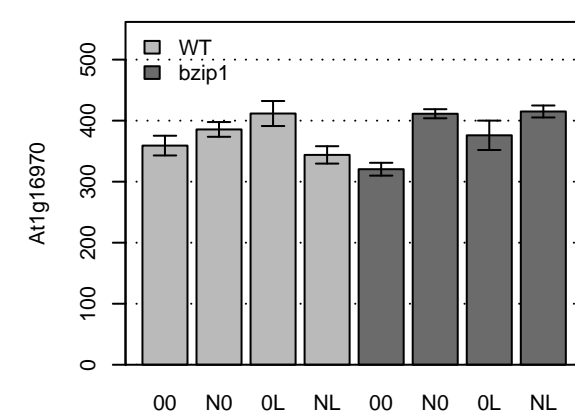

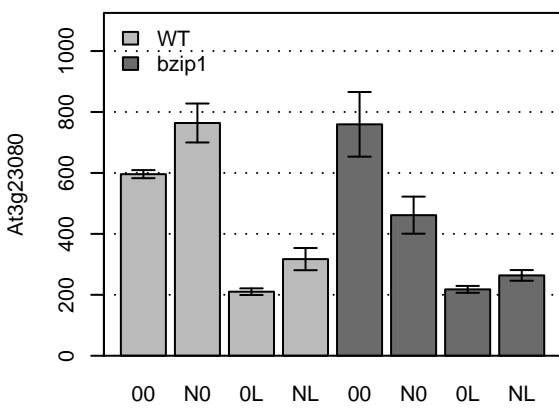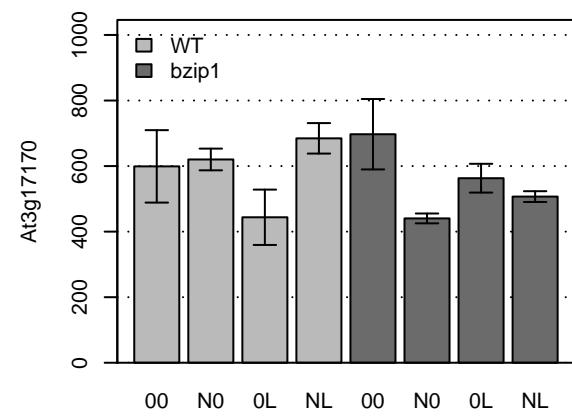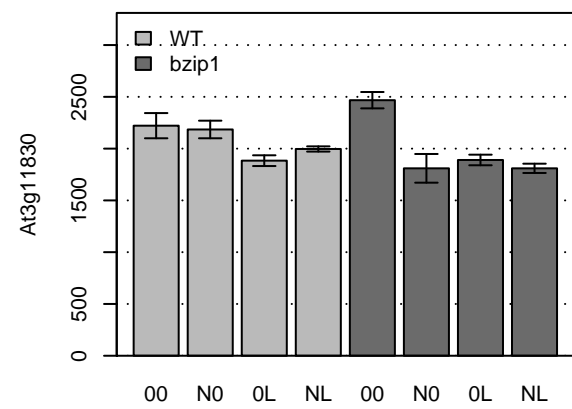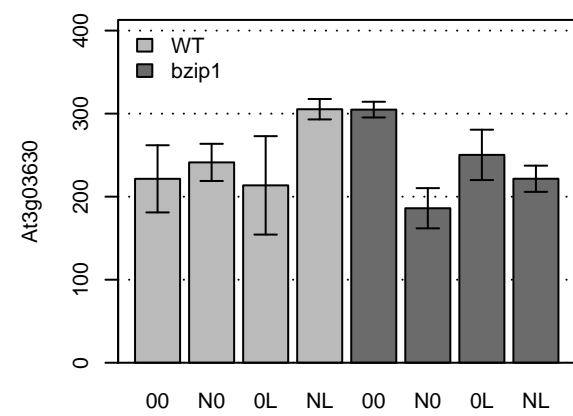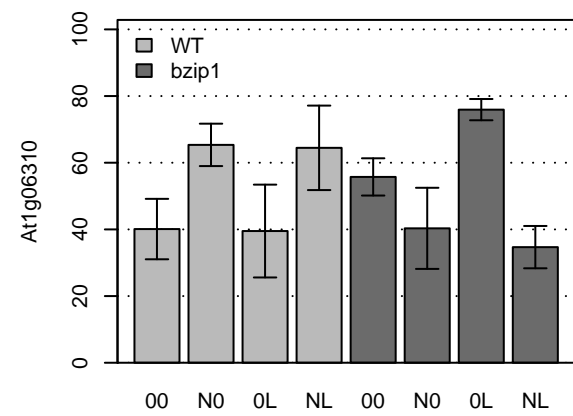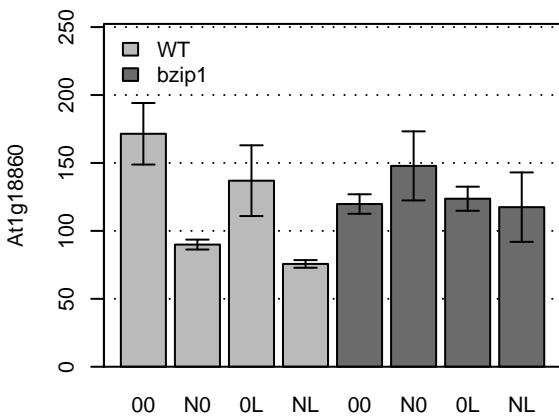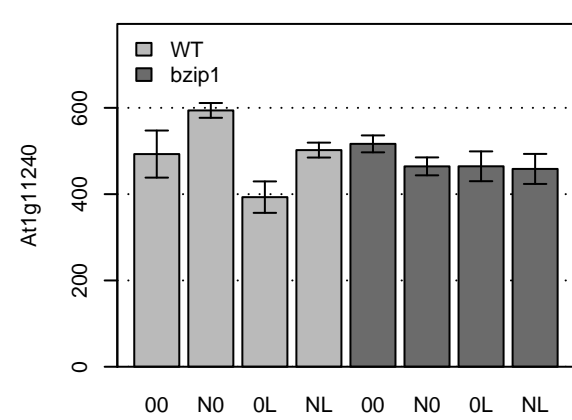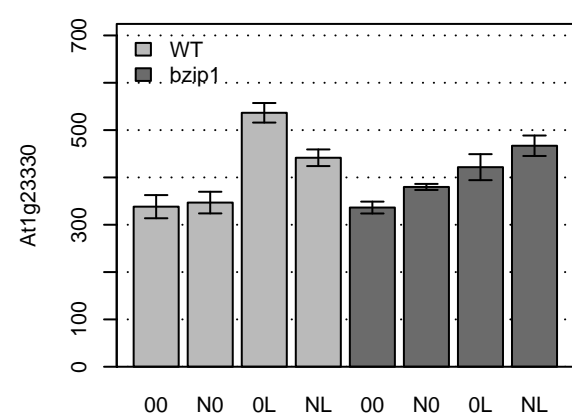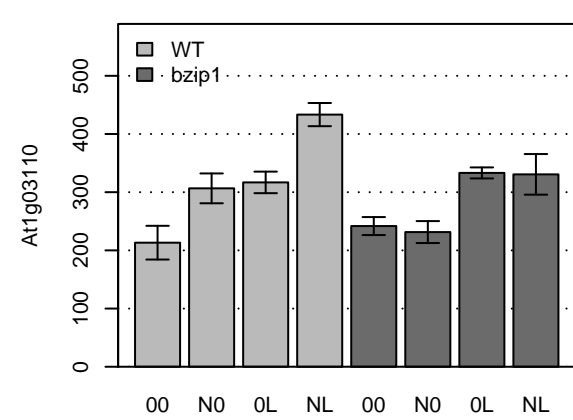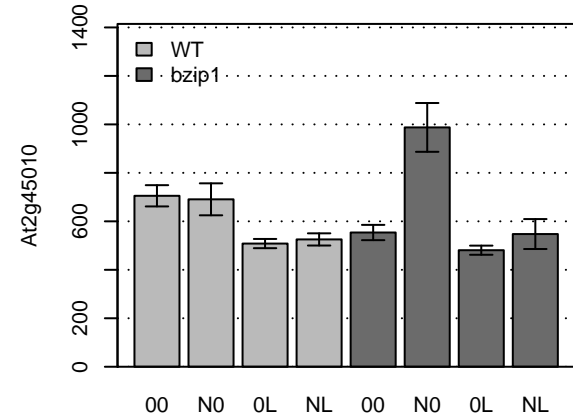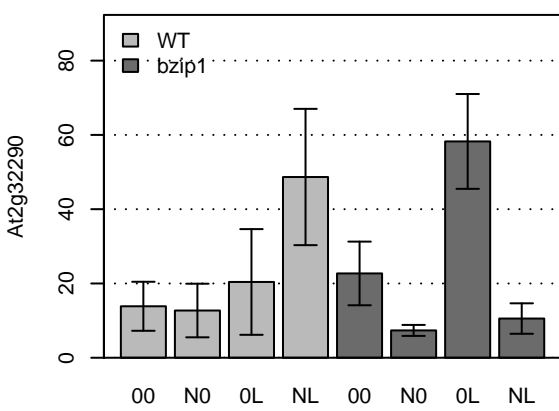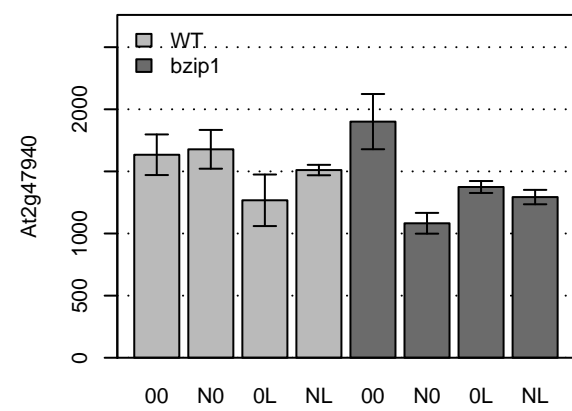

Supplement: Additional file 3 — Histograms show ATH1 array signals for the 219 genes regulated by genotype (bzip1-1 vs. WT sibling). The array data are averages of three biological replicates. Error bars = SE. [file 1752-0509-4-111-S3.PDF]
