# Supplementary material for: Modeling the global effect of the basic-leucine zipper transcription factor 1 (bZIP1) on nitrogen and light regulation in Arabidopsis
Source: BMC Syst Biol. 2010 Aug 12;4:111. doi: 10.1186/1752-0509-4-111 (PMC2933594; doi:10.1186/1752-0509-4-111)
Supplement: Additional file 4 — ANOVA output for each bZIP regulated gene cluster (for more details see Methods). [file 1752-0509-4-111-S4.DOC]

**Additional file 4:** Cluster ANOVA output.

**Cluster 1**

Df Sum Sq Mean Sq F value Pr(>F)

G1 1 4.1111 4.1111 93.5995 < 2.2e-16 ***

N1 1 0.0090 0.0090 0.2042 0.651644

L1 1 8.9041 8.9041 202.7225 < 2.2e-16 ***

G1:N1 1 0.2559 0.2559 5.8263 0.016336 *

G1:L1 1 0.3792 0.3792 8.6337 0.003534 **

N1:L1 1 0.0623 0.0623 1.4183 0.234538

G1:N1:L1 1 0.0145 0.0145 0.3299 0.566134

Residuals 328 14.4067 0.0439

---

Signif. codes: 0 '***' 0.001 '**' 0.01 '*' 0.05 '.' 0.1 ' ' 1

**Cluster 2**

Df Sum Sq Mean Sq F value Pr(>F)

G1 1 15.291 15.291 85.1321 < 2.2e-16 ***

N1 1 1.145 1.145 6.3758 0.012237 *

L1 1 1.359 1.359 7.5681 0.006410 **

G1:N1 1 0.408 0.408 2.2694 0.133315

G1:L1 1 0.534 0.534 2.9748 0.085903 .

N1:L1 1 0.805 0.805 4.4836 0.035287 *

G1:N1:L1 1 1.440 1.440 8.0162 0.005043 **

Residuals 232 41.670 0.180

**Cluster 3**

Df Sum Sq Mean Sq F value Pr(>F)

G1 1 0.2621 0.2621 10.1407 0.001673 **

N1 1 0.0712 0.0712 2.7528 0.098590 .

L1 1 0.2371 0.2371 9.1751 0.002764 **

G1:N1 1 0.0553 0.0553 2.1412 0.144895

G1:L1 1 2.2478 2.2478 86.9650 < 2.2e-16 ***

N1:L1 1 0.1056 0.1056 4.0874 0.044484 *

G1:N1:L1 1 0.0579 0.0579 2.2388 0.136104

Residuals 208 5.3761 0.0258

**Cluster 4**

Df Sum Sq Mean Sq F value Pr(>F)

G1 1 8.9611 8.9611 179.9397 < 2.2e-16 ***

N1 1 0.7337 0.7337 14.7325 0.0001408 ***

L1 1 0.6122 0.6122 12.2938 0.0004979 ***

G1:N1 1 0.1328 0.1328 2.6667 0.1031379

G1:L1 1 0.3498 0.3498 7.0246 0.0083097 **

N1:L1 1 0.0663 0.0663 1.3311 0.2492015

G1:N1:L1 1 0.8008 0.8008 16.0800 7.057e-05 ***

Residuals 472 23.5059 0.0498

**Cluster 5**

Df Sum Sq Mean Sq F value Pr(>F)

G1 1 0.622 0.622 4.3836 0.036848 *

N1 1 0.160 0.160 1.1273 0.288918

L1 1 29.247 29.247 206.1973 < 2.2e-16 ***

G1:N1 1 0.336 0.336 2.3670 0.124634

G1:L1 1 0.761 0.761 5.3619 0.021032 *

N1:L1 1 0.126 0.126 0.8912 0.345670

G1:N1:L1 1 1.086 1.086 7.6574 0.005888 **

Residuals 448 63.545 0.142

**Cluster 6**

Df Sum Sq Mean Sq F value Pr(>F)

G1 1 0.6194 0.6194 4.8840 0.028779 *

N1 1 2.5354 2.5354 19.9905 1.626e-05 ***

L1 1 3.2761 3.2761 25.8305 1.208e-06 ***

G1:N1 1 0.1816 0.1816 1.4321 0.233500

G1:L1 1 0.2407 0.2407 1.8982 0.170544

N1:L1 1 1.0123 1.0123 7.9818 0.005437 **

G1:N1:L1 1 0.7158 0.7158 5.6440 0.018911 *

Residuals 136 17.2487 0.1268

---

Signif. codes: 0 '***' 0.001 '**' 0.01 '*' 0.05 '.' 0.1 ' ' 1

**Cluster 7**

Df Sum Sq Mean Sq F value Pr(>F)

G1 1 6.0952 6.0952 115.2867 < 2.2e-16 ***

N1 1 0.1914 0.1914 3.6204 0.0580196 .

L1 1 1.1304 1.1304 21.3813 5.574e-06 ***

G1:N1 1 0.0019 0.0019 0.0369 0.8478324

G1:L1 1 0.4761 0.4761 9.0043 0.0029165 **

N1:L1 1 0.8218 0.8218 15.5448 0.0001001 ***

G1:N1:L1 1 0.0400 0.0400 0.7560 0.3852638

Residuals 304 16.0723 0.0529

**Cluster 8**

Df Sum Sq Mean Sq F value Pr(>F)

G1 1 8.7154 8.7154 217.3734 < 2.2e-16 ***

N1 1 1.4555 1.4555 36.3029 3.528e-09 ***

L1 1 0.0224 0.0224 0.5575 0.45566

G1:N1 1 0.0786 0.0786 1.9606 0.16214

G1:L1 1 0.1819 0.1819 4.5366 0.03372 *

N1:L1 1 0.0530 0.0530 1.3227 0.25072

G1:N1:L1 1 0.0221 0.0221 0.5506 0.45844

Residuals 448 17.9622 0.0401

**Cluster 9**

Df Sum Sq Mean Sq F value Pr(>F)

G1 1 0.4295 0.4295 11.4737 0.0007805 ***

N1 1 0.0027 0.0027 0.0721 0.7884540

L1 1 11.2704 11.2704 301.1134 < 2.2e-16 ***

G1:N1 1 0.0047 0.0047 0.1247 0.7241703

G1:L1 1 0.2376 0.2376 6.3490 0.0121581 *

N1:L1 1 0.2794 0.2794 7.4640 0.0065915 **

G1:N1:L1 1 0.0863 0.0863 2.3068 0.1296496

Residuals 376 14.0734 0.0374

**Cluster 10**

Df Sum Sq Mean Sq F value Pr(>F)

G1 1 3.3992 3.3992 89.0001 < 2.2e-16 ***

N1 1 0.1624 0.1624 4.2521 0.039847 *

L1 1 4.5272 4.5272 118.5335 < 2.2e-16 ***

G1:N1 1 0.0567 0.0567 1.4846 0.223770

G1:L1 1 0.3618 0.3618 9.4726 0.002229 **

N1:L1 1 0.0562 0.0562 1.4724 0.225688

G1:N1:L1 1 0.0286 0.0286 0.7501 0.386958

Residuals 400 15.2774 0.0382

**Cluster 11**

Df Sum Sq Mean Sq F value Pr(>F)

G1 1 0.4270 0.4270 5.0409 0.02613 *

N1 1 0.0121 0.0121 0.1428 0.70599

L1 1 0.0007 0.0007 0.0078 0.92951

G1:N1 1 0.0501 0.0501 0.5915 0.44298

G1:L1 1 4.6940 4.6940 55.4116 5.684e-12 ***

N1:L1 1 0.0982 0.0982 1.1597 0.28315

G1:N1:L1 1 0.3127 0.3127 3.6912 0.05648 .

Residuals 160 13.5538 0.0847

---

Signif. codes: 0 '***' 0.001 '**' 0.01 '*' 0.05 '.' 0.1 ' ' 1

**Cluster 12**

Df Sum Sq Mean Sq F value Pr(>F)

G1 1 0.3637 0.3637 10.0782 0.0018560 **

N1 1 0.4267 0.4267 11.8220 0.0007772 ***

L1 1 0.3960 0.3960 10.9733 0.0011843 **

G1:N1 1 0.0009 0.0009 0.0248 0.8750466

G1:L1 1 0.0661 0.0661 1.8315 0.1781998

N1:L1 1 0.1406 0.1406 3.8960 0.0504267 .

G1:N1:L1 1 1.5099 1.5099 41.8351 1.653e-09 ***

Residuals 136 4.9084 0.0361

**Cluster 13**

Df Sum Sq Mean Sq F value Pr(>F)

G1 1 0.6533 0.6533 7.9321 0.005387 **

N1 1 1.3462 1.3462 16.3457 7.752e-05 ***

L1 1 0.0120 0.0120 0.1453 0.703494

G1:N1 1 4.2602 4.2602 51.7256 1.557e-11 ***

G1:L1 1 0.0007 0.0007 0.0083 0.927675

N1:L1 1 0.4805 0.4805 5.8336 0.016701 *

G1:N1:L1 1 0.0464 0.0464 0.5632 0.453933

Residuals 184 15.1544 0.0824

**Cluster 14**

Df Sum Sq Mean Sq F value Pr(>F)

G1 1 0.1367 0.1367 1.2329 0.267995

N1 1 4.6357 4.6357 41.8127 5.875e-10 ***

L1 1 2.7204 2.7204 24.5372 1.404e-06 ***

G1:N1 1 0.8283 0.8283 7.4708 0.006754 **

G1:L1 1 2.7423 2.7423 24.7345 1.280e-06 ***

N1:L1 1 0.6411 0.6411 5.7826 0.016971 *

G1:N1:L1 1 0.1403 0.1403 1.2652 0.261835

Residuals 232 25.7215 0.1109

**Cluster 15**

Df Sum Sq Mean Sq F value Pr(>F)

G1 1 0.0001 0.0001 0.0014 0.9705888

N1 1 0.0009 0.0009 0.0106 0.9181615

L1 1 1.3421 1.3421 15.0478 0.0001366 ***

G1:N1 1 7.7971 7.7971 87.4206 < 2.2e-16 ***

G1:L1 1 0.7918 0.7918 8.8781 0.0031931 **

N1:L1 1 1.2774 1.2774 14.3219 0.0001962 ***

G1:N1:L1 1 0.0038 0.0038 0.0426 0.8367002

Residuals 232 20.6922 0.0892

**Cluster 16**

Df Sum Sq Mean Sq F value Pr(>F)

G1 1 0.0185 0.0185 0.3800 0.5381

N1 1 0.0119 0.0119 0.2430 0.6224

L1 1 0.0224 0.0224 0.4590 0.4986

G1:N1 1 6.5449 6.5449 134.1190 <2e-16 ***

G1:L1 1 0.0034 0.0034 0.0705 0.7908

N1:L1 1 0.0001 0.0001 0.0016 0.9678

G1:N1:L1 1 0.0005 0.0005 0.0111 0.9163

Residuals 304 14.8349 0.0488

---

Signif. codes: 0 '***' 0.001 '**' 0.01 '*' 0.05 '.' 0.1 ' ' 1

**Cluster 17**

Df Sum Sq Mean Sq F value Pr(>F)

G1 1 0.1906 0.1906 1.1042 0.2962339

N1 1 0.1189 0.1189 0.6888 0.4088040

L1 1 0.2410 0.2410 1.3964 0.2405135

G1:N1 1 1.0143 1.0143 5.8773 0.0173829 *

G1:L1 1 0.1085 0.1085 0.6287 0.4299754

N1:L1 1 2.3564 2.3564 13.6535 0.0003813 ***

G1:N1:L1 1 3.8130 3.8130 22.0938 9.537e-06 ***

Residuals 88 15.1873 0.1726

**Cluster 18**

Df Sum Sq Mean Sq F value Pr(>F)

G1 1 0.0378 0.0378 0.5587 0.456058

N1 1 0.3540 0.3540 5.2340 0.023692 *

L1 1 0.5548 0.5548 8.2032 0.004846 **

G1:N1 1 3.3997 3.3997 50.2700 6.631e-11 ***

G1:L1 1 0.1842 0.1842 2.7241 0.101151

N1:L1 1 0.0004 0.0004 0.0060 0.938436

G1:N1:L1 1 0.1477 0.1477 2.1839 0.141777

Residuals 136 9.1976 0.0676

**Cluster 19**

Df Sum Sq Mean Sq F value Pr(>F)

G1 1 0.1009 0.1009 0.8408 0.360560

N1 1 1.1176 1.1176 9.3130 0.002665 **

L1 1 0.0002 0.0002 0.0017 0.967026

G1:N1 1 0.1208 0.1208 1.0067 0.317204

G1:L1 1 0.0011 0.0011 0.0090 0.924636

N1:L1 1 0.0715 0.0715 0.5960 0.441241

G1:N1:L1 1 7.4767 7.4767 62.3065 4.387e-13 ***

Residuals 160 19.1998 0.1200

**Cluster 20**

Df Sum Sq Mean Sq F value Pr(>F)

G1 1 0.0538 0.0538 0.4976 0.48204

N1 1 0.2418 0.2418 2.2347 0.13776

L1 1 0.1603 0.1603 1.4820 0.22602

G1:N1 1 0.4077 0.4077 3.7685 0.05474 .

G1:L1 1 0.0180 0.0180 0.1668 0.68379

N1:L1 1 0.4748 0.4748 4.3886 0.03844 *

G1:N1:L1 1 5.9195 5.9195 54.7110 2.735e-11 ***

Residuals 112 12.1179 0.1082
